# Smart Magnetic Nanocarriers for Multi-Stimuli On-Demand Drug Delivery

**DOI:** 10.3390/nano12030303

**Published:** 2022-01-18

**Authors:** Parisa Eslami, Martin Albino, Francesca Scavone, Federica Chiellini, Andrea Morelli, Giovanni Baldi, Laura Cappiello, Saer Doumett, Giada Lorenzi, Costanza Ravagli, Andrea Caneschi, Anna Laurenzana, Claudio Sangregorio

**Affiliations:** 1 INSTM and Dipartimento di Ingegneria Industriale—DIEF, Università degli Studi di Firenze, 50139 Sesto Fiorentino, Italy; parisa.eslami@unifi.it (P.E.); andrea.caneschi@unifi.it (A.C.); 2 INSTM and Dipartimento di Chimica, U. Schiff, Università di Firenze, 50019 Sesto Fiorentino, Italy; 3 Dipartimento di Scienze Biomediche Sperimentali e Cliniche Mario Serio, Università degli Studi di Firenze, 50134 Florence, Italy; francesca.scavone@unifi.it (F.S.); anna.laurenzana@unifi.it (A.L.); 4 INSTM and Dipartimento di Chimica e Chimica Industriale, Università di Pisa, 56124 Pisa, Italy; andrea.morelli@unipi.it; 5 Ce.Ri.Col, Colorobbia Consulting S.R.L, 50059 Sovigliana-Vinci, FI, Italy; baldig@colorobbia.it (G.B.); cappiellol@colorobbia.it (L.C.); doumetts@colorobbia.it (S.D.); lorenzig@colorobbia.it (G.L.); ravaglic@colorobbia.it (C.R.); 6 ICCOM-CNR, 50019 Sesto Fiorentino, Italy

**Keywords:** magnetite nanoparticles, magnetic hyperthermia, drug delivery, thermo-responsive nanocarriers, pH-responsive nanocarriers, controlled drug release

## Abstract

In this study, we report the realization of drug-loaded smart magnetic nanocarriers constituted by superparamagnetic iron oxide nanoparticles encapsulated in a dual pH- and temperature-responsive poly (N-vinylcaprolactam-co-acrylic acid) copolymer to achieve highly controlled drug release and localized magnetic hyperthermia. The magnetic core was constituted by flower-like magnetite nanoparticles with a size of 16.4 nm prepared by the polyol approach, with good saturation magnetization and a high specific absorption rate. The core was encapsulated in poly (N-vinylcaprolactam-co-acrylic acid) obtaining magnetic nanocarriers that revealed reversible hydration/dehydration transition at the acidic condition and/or at temperatures above physiological body temperature, which can be triggered by magnetic hyperthermia. The efficacy of the system was proved by loading doxorubicin with very high encapsulation efficiency (>96.0%) at neutral pH. The double pH- and temperature-responsive nature of the magnetic nanocarriers facilitated a burst, almost complete release of the drug at acidic pH under hyperthermia conditions, while a negligible amount of doxorubicin was released at physiological body temperature at neutral pH, confirming that in addition to pH variation, drug release can be improved by hyperthermia treatment. These results suggest this multi-stimuli-sensitive nanoplatform is a promising candidate for remote-controlled drug release in combination with magnetic hyperthermia for cancer treatment.

## 1. Introduction

Cancer is considered as the top detrimental disease in the world. Chemotherapy is one of the most widely used treatments for various types of cancers; however, this method suffers from several drawbacks and significant limitations, the major one being the non-specificity of anticancer drugs, which may cause strong damage to healthy organs and cells during therapy.

Magnetic nanoparticles (MNPs) offer numerous advantages [1,2,3,4] including heat generation ability, lack of toxicity, high chemical stability, and good biocompatibility. All these properties confer to MNPs the potential to provide highly effective multimodal action for cancer diagnosis and treatment such as magnetic resonance imaging (MRI) [5,6,7], cell tracking [8,9], drug delivery [10,11,12,13,14], and magnetic fluid hyperthermia (MFH) [15,16,17,18,19]. The latter relies on destroying cancer cells by exploiting the heat released by MNPs upon excitation with an alternating magnetic field (AMF). The heat generated can raise the temperature of the surrounding environment in the temperature range from 42 °C to 48 °C, which causes apoptosis of cancer cells that are more sensitive to thermal damage than healthy cells [20,21,22]. In recent years, MFH has been investigated as a propitious tool in association with chemotherapy or radiotherapy [23,24,25]. Additionally, MFH has been studied as an adjuvant therapy with a drug delivery system to greatly augment the therapeutic efficacy of chemotherapeutic drugs at tumor sites [26,27]. This effect is due to improved intracellular uptake of drugs as a result of increased and leaky tumor vascularization and cell membrane permeability, inhibition of DNA repair, and acceleration of the cytotoxic chemical reactions [28,29,30,31,32]. Notwithstanding the advantages provided by combined magnetic hyperthermia and drug delivery, achieving efficient drug delivery vehicles with MNPs is still challenging owing to the lack of an appropriate controlled release system and possible leakage of conjugated drugs during circulation [33]. To solve these issues, smart organic–inorganic nanohybrids have been proposed to make multi-stimuli-responsive platforms able to deliver and discharge the drug at the right time and concentration by activation through external or internal stimuli such as temperature and pH [34,35].

Among the many approaches proposed in the literature, a particularly appealing one is the use of thermo-responsive drug nanocarriers, composed of a MNP core and a temperature-responsive polymer shell to promote the effective controlled release of the drug triggered by MFH [26,36,37,38]. Thermo-responsive, water-soluble polymers, such as poly (N-vinylcaprolactam) (PVCL), have been investigated in oncology to fulfill remotely controlled drug release [39,40]. Thermo-responsiveness is usually derived from a reversible conformational change from a swollen hydrophilic state to a shrunken hydrophobic one, occurring at the so-called lower critical solution temperature (LCST) [41,42,43,44]. Although thermo-responsive PVCL is more advantageous compared to other smart polymers used in biological application because of the higher biocompatibility and lower cytotoxicity, it has been scarcely considered for surface modification of MNPs on the grounds that the controlled radical polymerization of N-vinylcaprolactam monomers is inherently difficult [45,46,47,48].

The use of temperature-responsive polymers having the LCST in the hyperthermia temperature range (42–48 °C) is crucial for spatial and temporal control over the release of the drug, triggered by MFH [49]. To fulfil this goal, LCST of PVCL could be enhanced by including hydrophilic units such as poly acrylic acid (PAA). In fact, the presence of PAA segments modifies the hydrophilicity/hydrophobicity balance in copolymeric structure, leading to appropriate tuning of the LCST of poly (N-vinylcaprolactam-co-acrylic acid) (PVCL-co-PAA) at the desired temperature range, close to the therapeutic temperature range of MFH [50,51].

pH-responsive polymers such as poly acrylic acid (PAA) can also be utilized for stimuli-responsive drug delivery applications [52] based on significant pH variation between healthy and tumor tissues. The pH in the tumor extracellular matrix, in fact, is often more acidic than pH in normal tissues and blood, so if pH-sensitive drug nanocarriers target the tumor area, they can release their payload due to the pH difference [53]. Regardless of the fact that the pH is extensively used in endogenous on-demand drug delivery, its use has little potential to supply extremely precise and specific drug release at the target sites [54,55] owing to diminutive differences between normal and tumor tissues; thus, pH still needs to be combined with exogenous stimuli such as temperature to be more effective [56]. Recently, intelligent polymer sequences conferring sensitivity to more than one stimulus such as pH and temperature have been proposed as a unique opportunity in advanced nanocarrier-based drug delivery systems. This smart nanoplatform enables the simultaneous response to a combination of exogenous/endogenous stimuli, resulting in the further regulation of drug delivery and release, leading to reinforced in vitro/in vivo anticancer efficacy [37,57].

**Figure 1:**
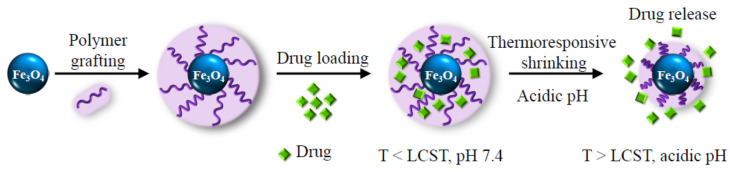
Schematic description of the smart magnetic nanocarriers, which offer drug loading capability and spatial and temporal control over the release of the drug.

In this context, our work was focused on the fabrication and characterization of smart magnetic nanocomposites, MNCs, comprising a superparamagnetic Fe_3_O_4_ MNP core and a dual pH- and temperature-sensitive PVCL-co-PAA shell conjugated with an anticancer drug (doxorubicin, DOX) for multimodal cancer therapy, by combining magnetic hyperthermia and controlled drug release, as schematized in Figure 1.

To this aim, MNPs with a specific flower-like morphology were prepared, which ensures a high magnetization and an extraordinary heat generation ability at their minimum concentration under low magnetic field amplitudes. Then, MNPs were efficiently encapsulated in the PVCL-co-PAA copolymer through a modified approach, which does not require using specific surfactant or cross-linker, yielding highly stable and effective smart multifunctional MNCs. As a matter of fact, PVCL and PAA segments on the surface of MNPs supply double pH- and temperature-responsive interfaces that imply unprecedented control over drug delivery and release in the biological system. Their relative ratio was properly chosen so as to tune the copolymer LCST in the hyperthermia range. We demonstrate that the resulting MNCs are biocompatible and can be loaded with a large amount of a cytotoxic drug, DOX, that was adopted as the model to investigate the drug release kinetics of the system both at physiological pH (7.4) and acidic pH (5.5, similar to that found in the tumor environment). More importantly, we show that the drug can be released on-demand by the application of an AMF with frequency and amplitude below the threshold for clinical application. This effect, in combination with the acidic pH of the endosomal compartment causes a significant reduction in tumor cells viability.

## 2. Materials and Methods

All chemicals of analytical grade were purchased from Sigma-Aldrich Co. Water was deionized and filtered with a Milli-Q System (Merck Millipore Co., Darmstadt, Germany). 2,2′-Azobis (2-methylpropionitrile) (AIBN, purity 98%) was recrystallized from methanol before use. N-vinylcaprolactam (NVCL, purity 98%) and acrylic acid (AA, purity 99%) were distilled under low pressure before copolymerization. 1,4-Dioxane anhydrous (purity 99.8%) was dried by sodium before use. All the other reactants and solvents were used with no further purification. Fe^0^ (Fe ≥ 99%, reduced fine powder), Iron (II) chloride (FeCl_2_, purity 98%), diethylene glycol (DEG, purity 99%), acetone (purity ≥ 99.9%), ethanol (EtOH, 99.8%), sodium hydroxide (NaOH, purity 99%), hydrochloric acid (HCl, 37 wt %), diethyl ether (purity 99%), doxorubicin hydrochloride (purity 98%), dialysis bag with molecular weight cut-off (MWCO) of 14 kDa, dialysis kit with MWCO of 1 kDa, Dulbecco′s Modified Eagle Medium (DMEM), and fetal bovine serum (FBS) were purchased from Sigma-Aldrich Co. MCF7 (breast cancer cell line) and A375 (human melanoma cell line) were obtained from the American Type Culture Collection (ATCC; Manassas, VA, USA). Acetic acid (CH_3_COOH, 80% vol.) was purchased from Thermo Fisher Scientific Co (Waltham, MA, USA).

### 2.1. Synthesis of (Fe_3_O_4_) Superparamagnetic Nanoparticles

Fe_3_O_4_ MNPs were prepared by the polyol approach. First, iron (III) acetate was prepared by mixing Fe^0^ (10.024 g, 0.179 mol) and 100.0 g of CH_3_COOH (1.67 mol) in the presence of H_2_O_2_ (11.73 mL, 0.5 mol) in 400.0 mL of DEG. Then, Fe (CH_3_COO)_3_ (46.6 g, 0.2 mol) was added to a solution of FeCl_2_ (12.675 g, 0.1 mol) in 130 mL of DEG under N_2_ flow. Afterward, precursors were mixed vigorously under reflux condition at 170 °C for 19 h under N_2_ atmosphere. Subsequently, the dark brown dispersion was cooled to room temperature and stored under N_2_. In order to increase the particle size of Fe_3_O_4_ MNPs and to provide a flower-like shape, the as-prepared dark brown suspension of MNPs in DEG was utilized as seeds, and reaction was similarly repeated with the same amount of precursors under reflux condition at 170 °C for 5 h. Final dispersion contains 1.33 wt % Fe_3_O_4_ MNPs, as evaluated from ICP-AES measurement.

### 2.2. Encapsulation of Fe_3_O_4_ in PVCL-co-PAA

PVCL-co-PAA was synthesized using the free radical polymerization approach, according to the slightly modified procedure outlined by Filip E Du Prez et al. [58] (Supplementary Materials Section S1 and Section S2, Figures S1–S3). The synthesized copolymer exhibits a sharp pH-dependent LCST above physiological body temperature (LCST ~39 °C at pH 5.5 and ~43 °C at pH 7.4, Supplementary Materials Section S2 and Figure S4) as determined by UV-Vis spectroscopy. For this purpose, Fe_3_O_4_ MNPs (27.93 mg, 0.12 mmol) suspended in 2.1 mL of DEG were precipitated by the addition of 50.0 mL acetone and then washed 3 times with ethanol/acetone to remove DEG completely. Then, PVCL-co-PAA (0.1178 g, 0.43 μmol, *M_n_* ~271,890 g mol^−1^, *M_w_* ~402,397.2 g mol^−1^, PDI ~1.48) was dissolved in 20.0 mL Milli-Q water by addition of 15.0 μL NaOH 0.1 M up to pH ~9 (the solubility of copolymer at neutral pH is too low due to the internal hydrogen bonds between acrylic acid groups in the polymeric backbone); afterward, pH was adjusted to 7.4 by addition of HCl 0.1 M and Fe_3_O_4_ MNPs were added to the polymer solution, sonicated for 1 h, and then stirred at room temperature overnight. Finally, functionalized MNPs were dialyzed with a dialysis bag (MWCO of 14 kDa) against buffer phosphate 10 mM, pH = 7.4 for 24 h. The final suspension is highly stable (sample A_7.4_, zeta potential (ζ) = −38.0 mV) and it contains 0.14 wt % MNPs as evaluated by ICP. The same procedure was applied to prepare a more concentrated suspension (20 mL) with high stability, containing 0.37 wt % MNPs (sample B_7.4,_ ζ = −40.0 mV), just by properly modifying the initial quantities of Fe_3_O_4_ MNPs (73.68 mg, 0.317 mol) and copolymer (0.3 g, 1.102 μmol). Samples A_5.5_ (0.14 wt % MNPs, ζ = −30.0 mV) and B_5.5_ (0.37 wt % MNPs, ζ = −33.0 mV) were prepared following the same procedure as A_7.4_ and B_7.4_ in buffer phosphate 10 mM at pH = 5.5.

### 2.3. Characterization Techniques

X-ray powder diffraction (XRD) measurement was carried out using a Bruker D8 Advance diffractometer equipped with Cu Kα (1.54178 Å) radiation and operating in θ−θ Bragg−Brentano geometry at 40 kV and 40 mA. The average particle size and morphology of uncoated MNPs and MNCs were examined by transmission electron microscopy (TEM) images recorded using a CM12 PHILIPS transmission electron microscope operating at 100 kV using a LaF6 source. Hydrodynamic diameter and surface charge value of MNP/MNC suspension were assessed by dynamic light scattering (DLS) analysis using a Nano-ZS Zetasizer (ZEN 3600-Malvern, Malvern, UK) equipped with a He–Ne laser (633 nm) at an angle of 173°. An equilibration time of 2 min was used before each measurement and at least five replicate runs (15 measurements each) were made for each sample. Temperature-responsive properties of the smart MNCs at acidic and neutral pH were evaluated by DLS on 1.0 mL of suspension containing 0.14 wt % and 0.37 wt % MNPs. The expected phase transition was observed by heating from 25 °C to 65 °C with a rate of 5 °C/min and equilibration time of ca. 10 min. The temperature at which the abrupt size increase occurred, was considered as the LCST value. Atomic emission spectrometer coupled with an inductively coupled plasma torch (ICP-AES, Varian 710-ES) was utilized for quantitative analysis of iron content in suspension, at wavelength of 238 nm. For this purpose, prior to analysis, all suspensions were digested with 2.0 mL of HNO_3_ and 2.0 mL of HCl. The FT-IR spectra of the MNPs, MNCs, and polymer were recorded with a double-beam Agilent Technology Cary 640 Series FT-IR spectrometer in the range of 4000–500 cm^−1^ using KBr pellets. Mass loss of a known quantity of MNCs as a function of temperature was monitored by thermogravimetric analysis (TGA) using a NETZSCH STA 449C Jupiter instrument. Measurement was carried out on about 50.0 mg of dried MNCs using an alumina sample-holder, and a scanning temperature range from 25 °C up to 1000 °C under gradual heating (5 °C min^−1^) under N_2_ atmosphere. The obtained result was compared with the mass loss of uncoated MNPs as reference (measurement recorded under the same conditions). Magnetic properties of MNPs and MNCs were evaluated by a Quantum Design MPMS SQUID magnetometer operating in the 1.8–350 K temperature range and with an applied field up to 50 kOe. Powder samples were hosted in a Teflon tape and then pressed in a pellet to prevent preferential orientation of the crystallites under the magnetic field. All data were corrected for the diamagnetic contribution of the Teflon and were normalized to the amount of magnetic material as evaluated from TGA. The field dependence of the magnetic moment (M vs. H) was measured cycling the field between ±50 kOe at 2.5 K and 300 K.

Calorimetric measurements of specific absorption rate (SAR) were performed using a 6.6 kW power supply by Fives Celes, France. The hyperthermic efficiency of Fe_3_O_4_ MNPs dispersed in DEG was measured by applying an alternating magnetic field (AMF) of ~21 kAm^−1^ amplitude (*H*_0_) and ~340 kHz frequency (*f*) on a 300 μL dispersion containing 1.33 wt % Fe_3_O_4_ MNPs for 140 s. The sample was placed in the mid-point of a copper coil and the temperature increase of the ferrofluid was measured as a function of time using an optical fiber temperature probe dipped in the MNPs’ dispersion. SAR was estimated from the heating profile according to the Equation (1)

(1) $$\mathrm{SAR}=\frac{m_{d}C_{p,d}}{m_{\mathrm{NP}}}{\left|\frac{dT}{dt}\right|}_{t\to 0}\;$$

where *m_d_* and *C_p,d_* are the mass and heat capacity of the dispersion medium (4186 and 2510 Jkg^−1^K^−1^ for water and DEG, respectively), and *m_NP_* is the mass of MNPs. Since the measurements are carried out in nonadiabatic conditions, *dT*/*dt* values were extrapolated for *t* → 0 by considering the initial slope of the temperature kinetic curve. To assess the influence of *f* and *H*_0_ on the heat dissipation ability of Fe_3_O_4_ MNPs, the SAR of the 1 mL of the same ferrofluid (1.33 wt % Fe_3_O_4_ MNPs) was also estimated using a 6 kW power supply by Fives Celes operating at lower *f* (183 kHz) and *H_AC_* (17 kA m^−1^), which are more suited for clinical application [59,60,61]. The hyperthermic efficiency of 1 mL of A_7.4_ and B_7.4_ MNC suspensions under exposure to an AMF with *f* = 183 kHz and *H*_0_ = 17 kAm^−1^ for 5 min was also evaluated.

### 2.4. Preparation of DOX-Loaded MNCs (DOX-MNCs)

Drug loading investigation was carried out on samples (A) (0.14 wt % MNPs) and (B) (0.37 wt % MNPs) to obtain DOX-MNC suspension with different MNP amounts and a fixed amount of DOX at various pH. Conjugation of DOX with the MNCs was performed by the addition of DOX (4.5 mg, 7.76 µmol) to 20.0 mL of buffer phosphate 10 mM suspension of MNCs at pH = 7.4 (samples A^DOX^_7.4_ and B^DOX^_7.4_) and pH 5.5 (samples A^DOX^_5.5_ and B^DOX^_5.5_), and the final DOX concentration was 0.225 mg mL^−1^. Then, the mixtures were incubated at 25 °C in the darkness for 72 h. After incubation, the DOX-MNC suspensions were dialyzed against buffer phosphate 10 mM, at pH = 7.4 or 5.5, using a dialysis membrane bag (MWCO ~1 kDa) for 24 h to remove the non-encapsulated drug. The concentration of free DOX in dialysis fluids was analyzed by UV-Vis spectroscopy at λ = 480 nm to determine the drug encapsulation efficiency (EE) and drug loading contents (DLC), using the following Equations (2) and (3)

(2) $$\mathrm{EE}\;(\%)=\frac{\mathrm{mass}\;\mathrm{of}\;\mathrm{total}\;\mathrm{drug}‒\mathrm{mass}\;\mathrm{of}\;\mathrm{unloaded}\;\mathrm{drug}}{\mathrm{mass}\;\mathrm{of}\;\mathrm{total}\;\mathrm{drug}}\times 100$$

(3) $${\mathrm{DLC}\;(µg\;\mathrm{mg}}^{-1})=\frac{\mathrm{mass}\;\mathrm{of}\;\mathrm{loaded}\;\mathrm{drug}}{\mathrm{mass}\;\mathrm{of}\;\mathrm{DOX}-\mathrm{MNC}}$$

Standards of DOX in buffer phosphate 10 mM at the selected pH were prepared to obtain calibration curve. Furthermore, in order to evaluate the drug loading capacity of MNC suspension at neutral pH, two more samples, A^+DOX^_7.4_ (0.14 wt % MNPs, DOX = 0.375 mg mL^−1^) and B^+DOX^_7.4_ (0.37 wt % MNPs, DOX = 0.375 mg mL^−1^) with final volume of 20.0 mL were prepared according to the same procedure, but by using a larger initial quantity of DOX = 7.5 mg, 0.013 mmol.

### 2.5. In Vitro Drug Release of DOX-MNCs without AMF

Typically, 3 samples of A^DOX^_7.4_ (5 mL) were separately inserted in a dialysis bag (MWCO ~14 kDa) and dialyzed under mild stirring against 50.0 mL of buffer phosphate at physiological pH (7.4) and acidic pH (~5.5 and ~4.5), respectively. Dialysis set up were either maintained to room temperature or placed into a preheated water bath at 37 °C (physiological temperature, < LCST) and 48 °C (hyperthermia temperature, > LCST). At predetermined time points (0.5, 1, 2, 3, 4, 5, 6, 7, 24, 30, 48, and 72 h), the dialysate (50.0 mL) was taken out, and 50.0 mL of fresh buffer phosphate with the appropriate pH was added to preserve sink conditions. Afterward, the volume of extracted dialysate was decreased to 5.0 mL by rotary evaporation, so that the absorbance of DOX released in the dialysis fluid came into the detectable range of UV-Vis spectroscopy at λ = 480 nm.

### 2.6. Drug Release under Continuous AMF

AMF-triggered drug release was measured continuously on 5.0 mL of sample A^DOX^_7.4_ or A^DOX^_5.5_ placed in a circulating bath at 37 °C. Measurements were carried out using a 6 kW power supply by Fives Celes for different time intervals (30, 40, 50, and 60 min) applying a 17 kA m^−1^ amplitude and 183 kHz frequency AMF. Then, the sample was taken out of the instrument and centrifuged at 7000 rpm for 15 min to separate the MNPs from the supernatant. The supernatant was analyzed by UV-Vis spectroscopy at λ = 480 nm for the evaluation of the amount of drug released.

### 2.7. Smart Hyperthermia with Switchable Drug Release

Switchable drug release experiment was carried out on the most concentrated samples B^DOX^_7.4_ and B^DOX^_5.5_ to better evaluate the effect of a controlled on-demand drug release under application of the AMF. To this aim, 5.0 mL of B^DOX^_7.4_ was placed in the copper coil, and then the AMF with *f* = 183 kHz and *H*_0_ = 17 kA m^−1^ was turned on for 3 min, allowing the MNC suspension to be heated. The AMF was then turned off for 10 min, and the sample allowed to cool down to 25 °C. This process was repeated 10 times. The DOX released during each cycle was collected after separation of the MNPs by centrifugation (7000 rpm, 15 min), and its absorbance at λ = 480 nm was measured by UV-Vis spectroscopy.

### 2.8. Cytotoxicity Assay and Cellular Uptake Study

Since DOX is one of the most effective anticancer drugs used for the treatment of solid tumors of diverse origins and in particular for breast cancer, MCF7 (breast cancer cell line) and A375 (melanoma cell line) were selected for the cytotoxicity tests and cellular uptake evaluation of drug nanocarriers. A375 and MCF-7 cell lines were grown in culture dishes as a monolayer in DMEM supplemented with 10% FBS, in a humidified atmosphere with 5% CO_2_. Cell viability of A375 cells against MNCs, free DOX, and DOX-MNCs was assessed by WST-1 assay. To this aim, cells were seeded on a 96-well plate at a density of 5.0 × 10^3^ cell/well and incubated at 37 °C under 5.0% of CO_2_ for 24 h. Then, cell culture medium was substituted with 100 μL of a fresh medium containing testing samples with different concentrations and incubated for 6 h. After incubation, the samples were removed, and 10.0 µL of cell proliferation reagent WST-1 was added to each well and incubated for 1 h. Then, the absorbance of samples was measured in a microplate reader at λ= 450 nm to calculate the viability of cells. In parallel, wells containing just cells with the culture medium were used as control, whereas wells with culture medium, MNCs, free DOX, or DOX-MNCs without cells were used as blank.

The viability of tumor cells was also determined by trypan blue staining. Cells (1.5 × 10^5^) were seeded in 6-well plates and allowed to attach overnight. On the next day, MNC colloidal suspensions were added at the indicated concentrations. Six hours later, cells were aseptically transferred to a 1.5 mL clear Eppendorf tube and incubated at room temperature or stimulated with AMF for 20 min. Then, 20 µL of cell suspensions were resuspended with an equal volume of 0.4% (*w*/*v*) trypan blue solution prepared in 0.81% NaCl and 0.06% (*w*/*v*) dibasic potassium phosphate. Viable and non viable cells (trypan blue positive) were counted separately using a dual-chamber hemocytometer and a light microscope. The means of three independent cell counts were pooled for analysis.

Confocal fluorescence microscopy was used to observe the intracellular uptake and distribution of DOX-MNCs. In total, 20.0 µL aqueous solution of DOX (0.58 mg mL^−1^, 1.0 mmol L^−1^) was added to 485.0 µL of sample A_7.4_ and the volume of the mixture was adjusted to 2.0 mL by addition of buffer phosphate 10 mM, pH = 7.4. Afterward, the mixture was incubated at 25 °C for 24 h, and then DOX-MNC suspension was dialyzed against buffer phosphate 10 mM, at pH = 7.4 by dialysis membrane bag (MWCO ~1 kDa) for 2 h. The final suspension contains 0.34 mg mL^−1^ Fe_3_O_4_ MNPs and 10 µmol L^−1^ DOX as evaluated by ICP and UV-Vis spectroscopy at λ = 480 nm, respectively. For cellular uptake evaluation, A375 and MCF7 cells were grown overnight in 18 mm glass coverslips with 10% DMEM for 24 h. The culture medium was replaced with DOX-MNCs (0.0058 mg mL^−1^ ~10.0 µmol L^−1^ DOX and 0.34 mg mL^−1^ Fe_3_O_4_) and then incubated 6 h. Finally, cells were fixed with 4% paraformaldehyde, and mounted with Prolong Antifade Mounting Medium on a clean glass slide. Slides were observed under a fluorescence confocal microscope FACSCAN LSRII (Becton–Dickinson, laser irradiation 530 nm).

## 3. Results and Discussion

### 3.1. Characterization of MNPs and MNCs

**Figure 2:**
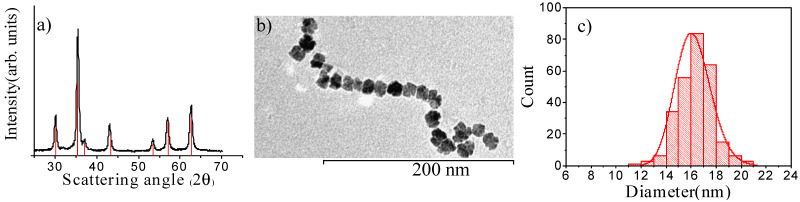
X-ray diffraction pattern of Fe_3_O_4_ MNPs prepared by polyol method compared to the reference pattern of magnetite (red bars; PDF 65-3107) (**a**); TEM image of Fe_3_O_4_ MNPs (**b**) together with the size distribution (**c**) obtained from a statistical analysis over ca. 280 MNPs. The continuous red line represents the best-fit curve to a log-normal distribution.

Fe_3_O_4_ MNPs were obtained by the polyol approach using DEG as solvent. The XRD pattern, reported in Figure 2a, confirms the sample consists of high crystalline magnetite MNPs (the measured lattice parameter, a = 8.399(1) Å is close to that expected for Fe_3_O_4_, 8.396 Å). In Figure 2b a representative TEM image of Fe_3_O_4_ MNPs is shown, and displays the sample consists of almost flower-like MNPs with a narrow size distribution and an average diameter of 16.4 ± 2.4 nm (d_TEM_) (Figure 2c); the dimension of an individual petal is estimated to be nearly 5–8 nm (Figure S6, Supplementary Materials).

Subsequently, Fe_3_O_4_ MNPs were encapsulated in the dual pH- and temperature-sensitive copolymer PVCL-co-PAA to provide smart MNCs. PVCL-co-PAA was synthesized following the free radical polymerization approach described by Filip E Du Prez et al. [58] (Supplementary Materials Sections S1 and S2). However, the procedure was modified to increase the length of PAA blocks and to enhance the number of carboxylic acid units. The latter grants a stronger interaction with the iron oxide surface via strong coordinate bonding without requiring the addition of any cross-linker and allows the conjugation of chemotherapeutics such as doxorubicin with excellent yield. The synthesized copolymer exhibits a sharp pH-dependent LCST above physiological body temperature (LCST ~39 °C at pH 5.5 and ~43 °C at pH 7.4; Supplementary Materials Section S3 and Figure S5), which makes this copolymer a suitable candidate for hyperthermia-controlled drug release.

**Figure 3:**
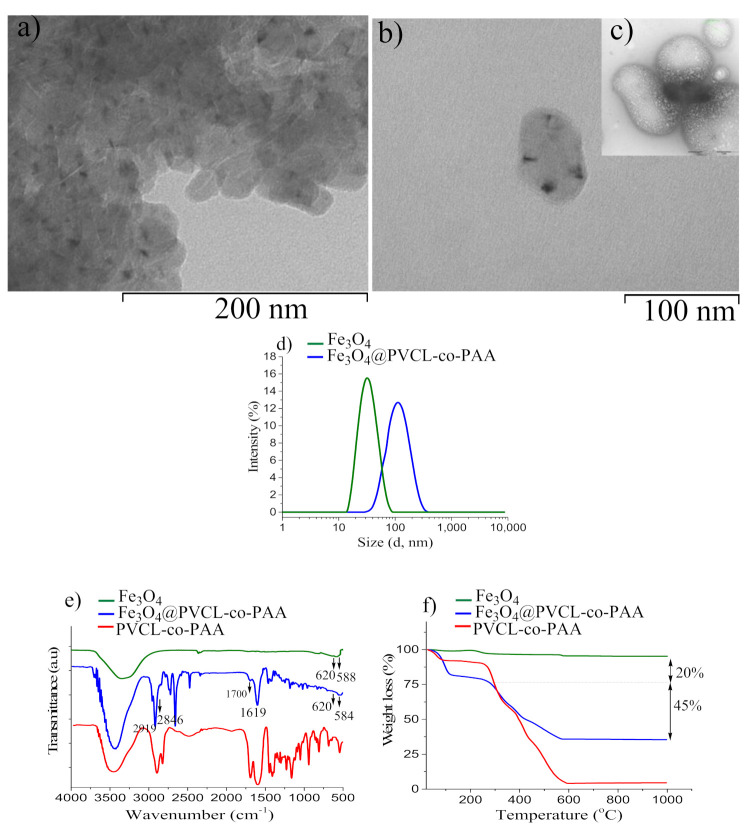
TEM images of Fe_3_O_4_@ PVCL-co-PAA (sample A) at low (**a**) and higher magnification (**b**); TEM image of polymer before MNPs’ inclusion (**c**) (the scale is the same as (**b**)); Hydrodynamic diameter of MNPs and MNCs determined by DLS (**d**); FT-IR spectra of Fe_3_O_4_@PVCL-co-PAA (blue line), PVCL-co-PAA (red line), and uncoated Fe_3_O_4_ (green line) (**e**); TG curve of MNCs made up of Fe_3_O_4_ coated PVCL-co-PAA (**f**). For comparison, the TG curves of uncoated MNPs and polymer alone are also shown.

To evaluate encapsulation efficiency, the morphology of Fe_3_O_4_@PVCL-co-PAA nanocarrier was investigated by TEM, as shown in Figure 3a,b. It can be observed that MNPs are distributed within the polymer matrix, and the MNCs (average size 70 × 100 nm) retained the oval shape of the pristine polymer without MNPs (Figure 3b,c). Furthermore, as reported in Figure 3d, DLS analysis exhibited that the sample consists of a uniform dispersion of MNCs with a narrow polydispersity index (PDI = 0.194) and an average particle diameter of 105.0 nm. Evidently, this value is much larger than the average hydrodynamic particle size of Fe_3_O_4_ MNPs (32.0 nm, PDI 0.124), which verifies that the encapsulation of the MNPs in the copolymer matrix was performed successfully. In order to control the stability of the MNC suspension, the zeta potential was measured and found to be equal to −38.0 mV, confirming that the suspension was highly stable at neutral pH; in fact, the negative surface charge is due to the presence of acrylic acid at the MNCs’ surface. Encapsulation of MNPs with PVCL-co-PAA copolymer was also demonstrated by FT-IR analysis, displayed in Figure 3e. For MNCs (blue line), two peaks are observed at 1700 cm^−1^ and 1619 cm^−1^, which are characteristic of the stretching vibration of HO-C=O (carboxylic) groups in the acrylic acid domain and amide carbonyl group (NHC=O) in the PVCL domain at the surface of MNPs. The same peaks are observed in the FT-IR spectrum of PVCL-co-PAA (red line) as well. The characteristic Fe–O stretching vibration bands are also revealed at 620 cm^−1^ and 584 cm^−1^, as clearly seen in the FT-IR spectrum of uncoated Fe_3_O_4_ (green line). Furthermore, the two peaks at 2846 cm^−1^ and 2919 cm^−1^, corresponding to the aliphatic C-H groups of a polymer shell, verify that the polymer successfully grafted on the surface of MNPs. The amount of PVCL-co-PAA bound on the Fe_3_O_4_ surface was assessed by TG analysis. As shown in Figure 3f, the TGA curve of MNCs exhibited two significant steps in weight loss: the first at 95 °C (~20%) can be attributed to the evaporation of residual water adsorbed on the surface, while the second sharp mass loss (~45%) occurs between 230 °C and 576 °C, which corresponds to the thermal decomposition of the polymer (246 °C to 591 °C, Figure 3f, red line). Similar results were also obtained for MNCs utilized to prepare suspensions B (Supplementary Materials, Figure S7).

**Figure 4:**
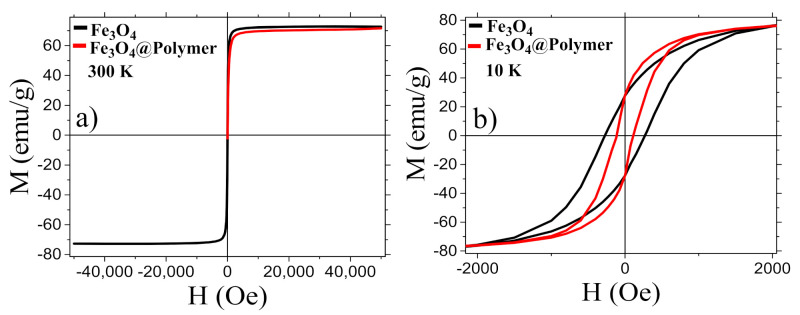
Room temperature field dependence of the magnetization of copolymer coated and uncoated MNPs measured in the field range ± 50 kOe (**a**) and enlargement of the low field region of the low temperature loop (**b**).

The field dependence of the magnetization of Fe_3_O_4_ MNPs and MNCs was investigated both at 300 K (Figure 4a) and 10 K (Figure 4b), and the principal magnetic parameters are summarized in Table 1. Both samples display a hysteretic behavior at low temperature; however, at the room temperature loop, no magnetic irreversibility was observed, indicating superparamagnetic-like behavior as expected for MNPs of this dimension. At both temperatures, the saturation magnetization, M_S_, is high, confirming the high crystallinity of the inorganic core. As reported in Table 1, the M_S_ and the remanence M_R_ of uncoated MNPs and MNCs at 300 K and 10 K are nearly equal for the two samples, and just a small difference is instead observed in the coercive field values that can be ascribed to the lower magnetic dipolar interaction between the magnetite cores in the coated samples due to the copolymer shell shielding [62,63,64].

The Fe_3_O_4_ MNPs (1.33 wt % Fe_3_O_4_ in DEG) displayed a very high SAR value of 613.0 W g^−1^_(Fe)_, (444.0 W g^−1^_(Fe3O4)_) under exposure to an AMF with frequency *f* = 340 kHz and amplitude *H*_0_ = 21 kA m^−1^ (Supplementary Materials, Figure S8). Under lower *f* (183 kHz) and *H*_0_ (17 kAm^−1^), below the threshold for clinical application, the average SAR value is still very remarkable, (357.0 W g^−1^_(Fe)_, 258.0 W g^−1^_(Fe3O4)_). These values are considerably higher than those commonly observed for spherical magnetite MNPs with similar volume [25,65]. This effect can be ascribed to the almost flower-like morphology of Fe_3_O_4_ MNPs achieved with the polyol approach. Indeed, nanoflowers are composed of highly ordered nanocrystals that do not behave like isolated grains [66] and do exhibit lower anisotropy due to coalescence and crystal ordering of the individual grains [67]. Conversely, spherical particles display higher surface disorder and, consequently, higher surface anisotropy. Altogether these factors make the MNPs behave as more efficient heat mediators for magnetic fluid hyperthermia. The hyperthermic efficiency of samples A_7.4_ and B_7.4_ was also evaluated when applying an AMF of *f* = 183 kHz and *H*_0_ = 17 kAm^−1^, and the temperature kinetics are reported in Supplementary Materials, Figure S9. A large temperature increase for both samples is remarkably observed, and the two suspensions reaching 43 °C (A_7.4_) and 71 °C (B_7.4_), respectively, after 5 min of field application. In both cases, the heating power is potentially sufficient to induce in vivo controlled drug release at the hyperthermia temperature range. In fact, the two suspensions, when applying an AMF with *H·f* below the critical threshold for clinical application, can reach a temperature above the LCST of the polymer.

### 3.2. Dual pH- and Temperature-Responsive Behavior of MNCs

**Figure 5:**
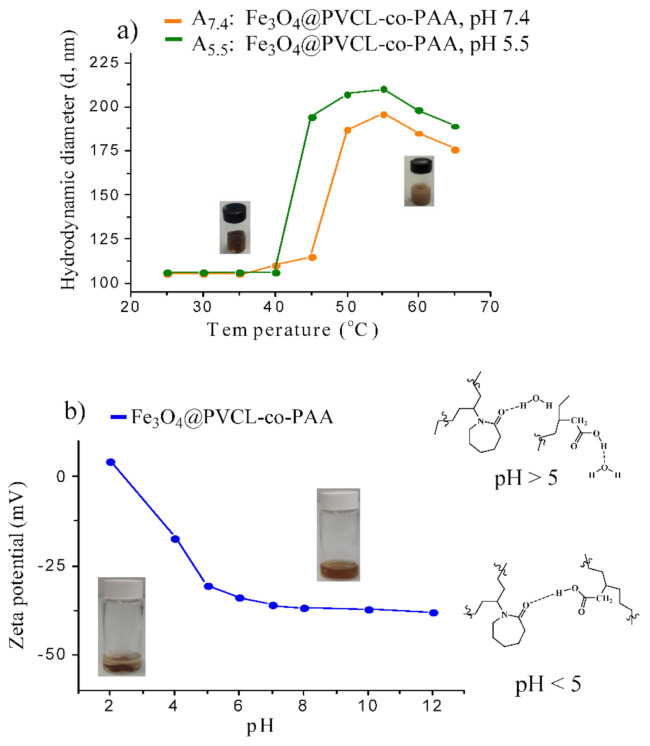
Hydrodynamic size variation of MNCs as a function of temperature, evaluated by DLS (**a**); zeta potential of MNCs as a function of pH measured by the dispersion of stimuli-responsive MNCs in buffer phosphate 10 mM; starting pH was 7.4 and was then adjusted with NaOH 0.1 M or HCl 0.1 M (**b**).

The temperature-responsive behavior of MNCs was investigated by DLS, both at physiological and tumor pH, on two buffer suspensions of MNCs (samples A_7.4_ and A_5.5_). The temperature at which the abrupt size increase occurred, was considered as the LCST. The results, shown in Figure 5a, exhibit the hydrodynamic size of stimuli-responsive nanocarrier suspension is highly temperature-dependent: below 40 °C (T < LCST) at both pH, the particle size of the suspension was almost constant (~105 nm), demonstrating that the polymer adhered strongly on the MNPs’ surface, and prevented aggregation. At T ≥ 45 °C (pH = 7.4) and at T ≥ 40 °C (pH = 5.5), the particle size was strongly enhanced (~187 nm at pH = 7.4 and ~194 nm at pH = 5.5), specifying that LCST was reached. Actually, at a temperature below LCST, hydrophilic copolymer chains stabilized MNCs in buffer phosphate solution through steric and electrostatic repulsion. When the T increased above LCST, the hydrophilic structure transformed into a hydrophobic structure, and due to the loss of steric repulsion, composite nanoparticles formed big aggregates, leading to a sharp increase in the particle size. For higher temperature (≥55 °C), a slow decrease in the particle size was observed since larger aggregates dissociate into smaller MNCs as a result of the crowding effect. These results agree with those obtained by UV-Vis spectroscopy on the copolymer solution alone (Supplementary Materials Section S2 and Figure S4), which indicates LCST of 39 °C and 43 °C at pH = 5.5 and 7.4, respectively. LCST of samples B_7.4_ and B_5.5_ was also found at the same value of the corresponding suspensions (A) as determined by DLS measurement (Supplementary Materials and Figure S10).

In order to check the pH-responsive behavior of the nanosystem, the zeta potential of A_7.4_ was measured as a function of pH at 25 °C, and the results are reported in Figure 5b. Stimuli-responsive nanohybrids are highly stable from pH = 5.0 to pH = 12.0 with large negative surface charge (from −33.0 to −38.0 mV). From pH = 5.0 to pH = 7.4, the stability of the MNC suspension enhanced due to the deprotonation of carboxylic acid units of PVCL-co-PAA. Higher pH values led to the ionic dissociation of the carboxylic acid of nanohybrids and resulted in fewer interactions between the acrylic acid (AA) and N-vinylcaprolactam (NVCL) units of the copolymer, providing higher stability of MNCs. As pH reduced below 5.0, the dispersion started losing stability and complete precipitation occurred as a consequence of protonation of carboxylic groups, which caused small positive surface charge, +4.0 mV. The lack of water dispersion of the MNCs is attributed to the formation of hydrogen bonding between the amide groups of NVCL and carboxylic moieties of copolymer on the surface of MNPs. However, by a simple addition of NaOH solution, clear and stable dispersion can be reobtained by shaking or sonication. Consequently, these results clearly emphasized a dual pH- and temperature-dependent behavior of MNCs, suggesting PVCL-co-PAA can be exploited to control the delivery of drugs in specific organs or intracellular compartments (endosomes or lysosomes), which in principle allows for tailored drug release with excellent spatial, temporal, and dosage control when subtle environmental changes are associated with pathological situations such as cancer or inflammation.

### 3.3. Drug Loading and Release

To evaluate the drug loading potential of stimuli-responsive MNCs, a cationic chemotherapeutic drug, DOX, with broad-spectrum antitumor activity [68], was selected. The efficiency of the loading of DOX on MNCs was assessed on 20.0 mL of MNC suspensions at pH 7.4 (samples A^DOX^_7.4_, B^DOX^_7.4_, A^+DOX^_7.4_, and B^+DOX^_7.4_) and pH 5.5 (samples A^DOX^_5.5_, B^DOX^_5.5_), and the loading data are summarized in Table 2. The encapsulation efficiency (EE) of sample A_7.4_ is 90% at neutral pH, ca. 2 times higher than that obtained for sample A^DOX^_5.5_, 45.0%; similarly, the EE of sample B^DOX^_7.4_ is 92.0%, almost 1.7 times more than the EE of sample B^DOX^_5.5_ (53.0%).

**Figure 6:**
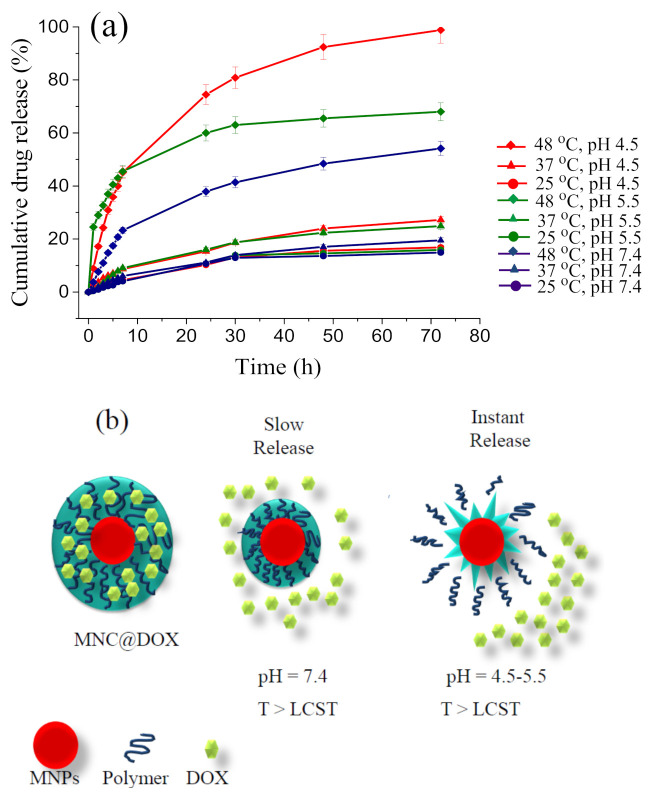
Release of DOX from DOX-MNCs at different pH values (7.4, 5.5, and 4.5) and temperatures (25 °C, 37 °C, and 48 °C); Standard deviation of triplicate drug release tests (*n* = 3) (**a**); Scheme of the drug release mechanisms operating in MNCs: gentle release caused by shrinkage of the temperature-responsive polymer under heating at neutral pH and intense release at pH 4.5 and 5.5 due to additional ruptures of the nanocarrier (**b**).

The results suggest the extraordinary capability of smart MNCs to load high contents of DOX at neutral pH. In fact, their high negative surface charge at physiological pH favors electrostatic interactions with positively charged DOX (pKa = 8.3) and enhances EE%. Furthermore, attractive interactions between the aromatic rings of DOX and the carbonyl groups in the structure of PVCL-co-PAA-based MNCs amplify the interaction between DOX and the MNCs’ surface to enhance EE%. On the other hand, at acidic pH, EE and DLC decreased due to the protonation of polyacrylic acid in the structure of the copolymer shell, resulting in repulsive electrostatic force between the positive surface charge of DOX and the copolymer shell. Importantly, the EE of samples (B) are higher than those of the corresponding samples (A) since higher polymer quantity implies more PAA units available for electrostatic interaction with positively charged DOX. The EE of DOX-MNCs can also be further enhanced at neutral pH by increasing the initial DOX concentration in the suspension. For instance, by using DOX with the concentration of 0.375 mg mL^−1^ in 20.0 mL of suspension, an outstanding drug loading efficiency of 98.0% and 96.0% with DLC of 91.10 and 28.9 μg mg^−1^ for samples A^+DOX^_7.4_ and B^+DOX^_7.4_, respectively, were obtained, approving the promising potential of MNCs to carry high payloads of cationic drugs. To assess the efficiency of pH- and temperature-responsive MNCs for stimuli-controlled drug release, the cumulative DOX release was determined by dialysis technique at different temperatures and various pH, mimicking physiological (7.4), tumor microenvironment (5.5), and intracellular tumor endosome/lysosome (4.5)), and results are reported in Figure 6a. Analysis of the curves reveals that only a very low amount of DOX (<7.0%) was released at 25 °C and 37 °C during the first 7 h, independently of pH. However, at 48 °C, above LCST, the drug nanocarriers exhibit an increase in the released DOX (ca. 24.0% at pH = 7.4, and 45.0% at pH ~5.5 and 4.5) after 7 h. A similar behavior is also observed when a longer period of 72 h is considered: a maximum of 15.0% (at 25 °C), 19.5% (at 37 °C), and 54.0% (at 48 °C) of DOX was released under physiological pH, when only the temperature stimulus triggered the drug release. Indeed, the temperature-dependent reversible conformational change of copolymer from a swollen hydrophilic state to a shrunken hydrophobic one results in a volume decrease of MNCs, which triggers drug release in a controlled manner (Figure 6b). At pH 5.5, after 72 h, a maximum drug release of 15.9% and 24.8% was obtained for 25 °C and 37 °C, respectively. However, this value sharply increased up to 68.0% at 48 °C (above LCST), when the pH- and thermo-responsive polymer shrinks, squeezing the drug out from the nanocarrier. Finally, at pH = 4.5, an almost complete release of the conjugated drug (98.8%) is observed at 48 °C after 72 h, when the DOX-MNCs were exposed to both pH- and temperature-stimuli. This effect is owing to the collapse of the nanocarrier, as schematized in Figure 6b. This remarkable pH- and temperature-triggered drug release property of the MNCs can be explained by several reasons: on one hand, the lack of water solubility of the copolymer at acidic pH (Figure 5b), and enhanced temperature above LCST, leading to the additional rupture of the nanocarrier and higher drug release efficiency (Supplementary Materials, Figure S5); on the other hand, at acidic pH, the increased protonation of the carboxylic acid units of the PVCL-co-PAA nanocarrier leads to a reduction in the electrostatic attraction with DOX (at acidic pH, the primary amine group of DOX is protonated, pKa = 8.3), increasing the drug release efficiency. Moreover, the protonation of DOX at acidic pH increases its solubility in an aqueous medium, facilitating its release out of the copolymer layer. Overall, the excellent loading and release properties of the designed PVCL-co-PAA-based magnetic nanocarriers confer an excellent potential on MNCs to be employed as efficient drug encapsulation and delivery systems with the capability of controlled pH and temperature on-demand drug release on acidic tumor microenvironments in combination with hyperthermia performance.

### 3.4. Drug Release under the Application of an AMF

**Figure 7:**
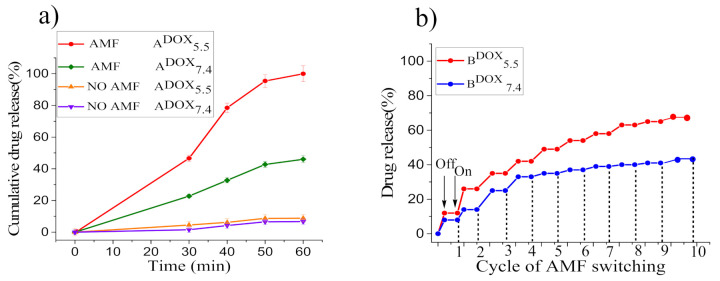
Cumulative DOX release of A^DOX^_7.4_ and A^DOX^_5.5_ for different exposure times to the AMF. Standard deviation of triplicate drug release tests (*n* = 3) (**a**); on–off switching cycles of AMF and DOX release profile corresponding to reversible swell–shrink property of samples B^DOX^_7.4_ and B^DOX^_5.5_ when applying an intermittent AMF (**b**).

The achievement of highly effective drug release from smart MNCs at the tumor site, in addition to an endogenous stimulus (pH), requires that MNPs’ core can induce a temperature increase through magnetic hyperthermia under applying an AMF. Hence, cumulative DOX release of samples A^DOX^_7.4_ (LCST ~45 °C) and A^DOX^_5.5_ (LCST ~40 °C) when applying an AMF with *f* = 183 kHz and *H*_0_ = 17 kAm^−1^ was estimated as a function of time and results are shown in Figure 7a and Supplementary Materials, Figures S11 and S12; moreover, in order to verify that the enhanced drug release occurred as a consequence of the localized heating caused by the MNPs under AMF excitation, a control drug release experiment was set up at 37 °C without AMF, and, even under this condition, a low drug release percentage was assessed after 60 min at both pH (6.7% at pH = 7.4 and 8.8% at pH = 5.5). Conversely, significant drug release was achieved at hyperthermia temperature (55 °C > LCST of the copolymer) for both samples (A^DOX^_7.4_, A^DOX^_5.5_) after 30 min under AMF exposure. Indeed, 23% of DOX at pH = 7.4 and 46.6% of DOX at pH = 5.5 were released. After 60 min of AMF exposure, the maximal drug release became 46.0% at pH = 7.4, while almost total release (99.98%) occurred at pH = 5.5 (maximum T reached 57 °C) [37]; thus, under AMF exposure the cumulative drug release of smart MNCs at pH = 7.4 and pH = 5.5 was, respectively, about 4.7 and 5.8 times greater than that obtained without the magnetic hyperthermia stimulus at 37 °C. Consequently, we can conclude that the combined effect of acidic pH and magnetic hyperthermia is highly advantageous for controlled drug release in the tumor site.

### 3.5. Smart Hyperthermia with Switchable Drug Release

A critical requirement for fully exploiting the potential of stimuli-responsive MNCs, is their capability to release drugs only when the external stimulus is active (switchable on/off drug release); to this aim, we verified the possibility of a controlled AMF-triggered drug release by repeatedly applying ‘on–off’ switches of AMF (*f* = 183 kHz and *H_AC_* = 17 kAm^−1^) for the overall application time of 30 min (3 min of AMF application, 10 cycles). Hyperthermia switchable drug release was performed on samples with the highest MNP concentration, B^DOX^_7.4_ (LCST = 45 °C, SAR = 453.0 W g^−1^_(Fe)_) and B^DOX^_5.5_ (LCST = 40 °C, SAR = 427.0 W g^−1^_(Fe)_), which display remarkably high hyperthermia performance and allow increasing temperature up to 53–58 °C in the very short time of application of the AMF (Supplementary Materials, Figures S13 and S14), and the obtained results are revealed in Figure 7b.

From the first to the tenth cycles of AMF exposure, DOX is released up to ca. 41.0% at pH = 7.4 and 65.0% at pH = 5.5, and no release occurs when the AMF is switched off. These results demonstrate that at the ‘on’ state at pH = 7.4 the MNCs’ deformation, which triggered drug release, depends only on the temperature variation induced by magnetic hyperthermia. Conversely, at pH = 5.5 at the ‘on’ state, the acidic pH stimulus boosts drug release in combination with magnetic hyperthermia that extremely enhances the drug release efficacy. On the other hand, when the magnetic field is ‘off’, temperature reduces and the DOX release process is stopped, since the deformed copolymer grafted on MNCs’ surface absorbs water and re-swells to the hydrophilic phase. This result clearly demonstrates the temperature reversibility of the drug release process from our smart MNCs, which can be exploited for realizing ‘‘on/off’’ drug delivery systems by using the trigger induced by AMF excitation with low *f* and *H_0_* and within a short period of time (~30 min).

### 3.6. Cytotoxicity Investigation and Cellular Uptake

**Figure 8:**
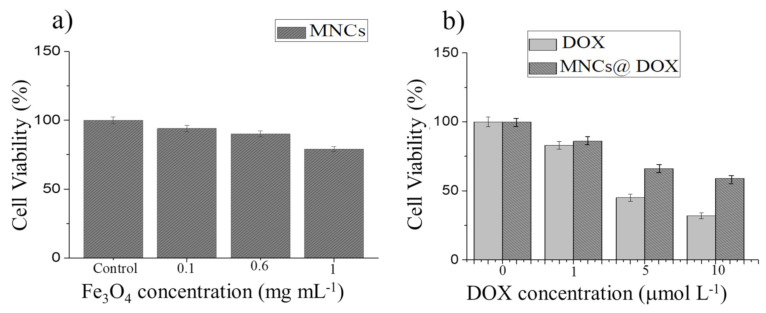
Relative cell viabilities of A375 human melanoma cells incubated with different concentrations of MNCs (**a**), free DOX and DOX-MNCs (**b**) during 6 h incubation.

Since MNPs, as therapeutic agents, must be nontoxic, cytotoxic effects of MNCs (1.0, 0.6, and 0.1 mg mL^−1^ of Fe_3_O_4_ MNPs), free DOX (10.0, 5.0, and 1.0 µmol L^−1^), and DOX-MNCs (DOX = 10.0, 5.0, and 1.0 µmol L^−1^ at concentration of MNPs = 0.1 mg mL^−1^) were investigated on A375 human melanoma cells by WST-1 assay over 6 h, and the results are shown in Figure 8. In Figure 8a, an in vitro cytotoxicity assessment of MNCs revealed a high cell viability (~94.0% and 90.0%) after 6 h of incubation with the sample concentration as high as 0.1 mg mL^−1^ and 0.6 mg mL^−1^, respectively, confirming the negligible toxicity and excellent biocompatibility of the MNCs. However, the cell viability of MNCs slightly decreased to 80.0% at the highest concentration (1.0 mg mL^−1^) after 6 h. As displayed in Figure 8b, in the DOX concentration range 1.0–10.0 µmol mL^−1^, the antitumor activity of the DOX-MNCs (viability 86.0%, 66.0%, and 60.0%) is lower than that of free DOX, in particular at the higher dose since in the absence of external stimuli (AMF), free DOX diffusion is much quicker than DOX release from the smart MNCs, which is only triggered by the acidic pH of the tumor compartment; therefore, these data demonstrate that without applying exogenous stimuli, DOX-MNCs have lower toxicity with respect to free DOX, verifying that the drug is actually encapsulated in the stimuli-responsive copolymer.

**Figure 9:**
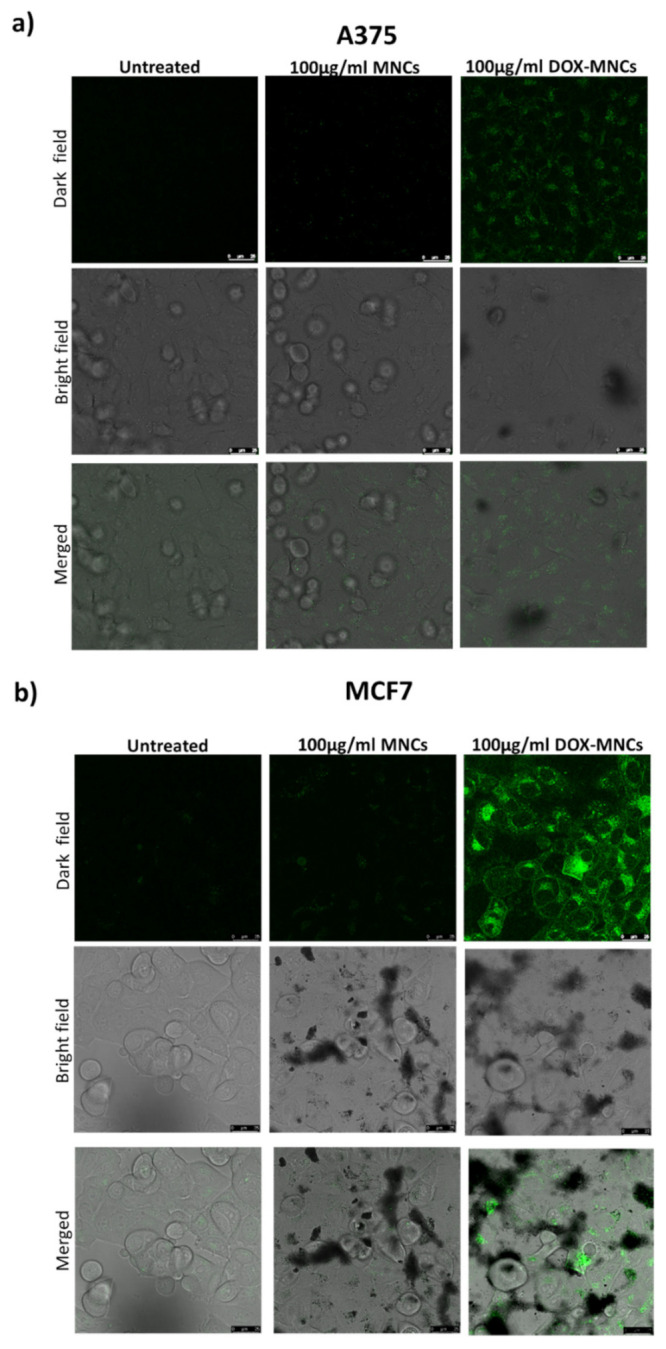
Confocal microscopy images of A375 melanoma (**a**) and MCF7 breast cancer cells (**b**) treated with vehicle (Control), MNCs, or DOX-MNCs for 6 h. The first row represents DOX fluorescent images, the second row the phase contrast microscopy images, and third row represent DOX overlay and merged images.

Based on these results, we selected a DOX-MNC suspension with 0.1 mg/mL^−1^ Fe_3_O_4_ and 1.0 µmol L^−1^ DOX concentrations for further investigation on the cellular uptake and efficacy of magnetic field-controlled release. The stability of this suspension and of that obtained by suspending the drug unloaded MNCs at the same concentration, was tested in DMEM supplemented with 10% or 20% FBS by monitoring the size evolution by DLS over 48 h. Despite a small increase in the average size observed at t = 0 (216 ± 14 and 200 ± 9 for MNC at 10% and 20% FBS, and 148 ± 9 and 241 ± 12 for DOX-MNC at 10% and 20% FBS, respectively) no aggregation occurred during the considered time span (Supplementary Materials, Figure S15). For this purpose, A375 human melanoma cells and MCF7 breast cancer cells were incubated at 37 °C with a DOX-MNC suspension for 6h and analyzed by fluorescence microscopy, allowing the DOX to be located as it emits green fluorescence under laser irradiation. The MCF7-treated cells (Figure 9b) show a brighter green fluorescence than the A375 cells (Figure 9a), indicating that conjugated MNPs are taken up more efficiently into the MCF7. It is also apparent that the fluorescence is concentrated in the cytoplasm, in accordance with the previous reports [69].

**Figure 10:**
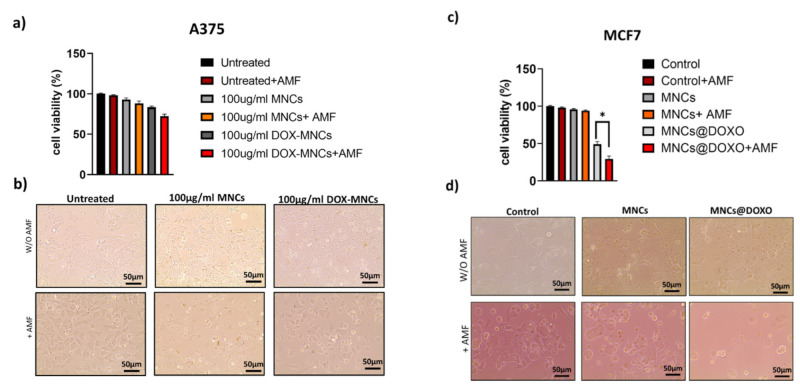
Relative cell viability and optical images of (**a**,**b**) A375 and (**c**,**d**) MCF7 cancer cells treated with vehicle (Control), MNC, or MNC@DOX for 24 h before and after AMF exposure. Error bars: mean ± SD. Statistical analysis was performed using one-way ANOVA followed by Newman–Keuls multiple comparison test. * *p* ≤ 0.05.

Then, we carried out in vitro cytotoxicity of unloaded and DOX-loaded MNCs before and after AMF application in A375 and MCF7 (Figure 10). Cells were treated as described above with 0.1 mg/mL^−1^ Fe_3_O_4_ or DOX-MNCs (0.1 mg/mL^−1^ Fe_3_O_4_ and 1.0 µmol L^−1^ DOX) for 24 h and then exposed to the AMF. The results reported in Figure 10a,c show that unloaded MNCs are nontoxic in both cell lines exhibiting an excellent biocompatibility, while the cell viability was slightly lowered after AMF stimulation. Concerning the therapeutic efficacy of DOX-loaded MNCs, Figure 10c shows that DOX-MNCs inhibit the viability of MCF7 with a remarkable enhancement of the cell death after AMF exposure due to the higher release rate of doxorubicin. It is to be noted that the mild effects of the MNCs containing DOX on A375 cell viability (Figure 10a), indicating their lower incorporation into the melanoma cells compared to MCF7 cell as shown in Figure 9a. In both cases, the cell viability was significantly reduced after AMF exposure, in particular from 54% to 35% for MCF7 cells and from 80% to 70% for A375 cells. These results confirm that the payload is continuously released from MNCs, triggered by the acidic pH of the endosomal compartment, while the DOX release can be boosted when an AMF is applied. The biological effects of an unloaded and loaded nanoparticle before and after AMF stimulation was further confirmed by morphological analysis of the cell cultures (Figure 10b,d).

## 4. Conclusions

The experimental work presented here was focused on the design of a novel smart magnetic nanocarrier, constituted by a nanometric Fe_3_O_4_ core encapsulated in a stimuli-responsive polymeric shell for controlled drug delivery in combination with magnetic hyperthermia for cancer therapy. To realize this goal, flower-like MNPs with an average diameter of ca. 16.4 nm (each petal measuring 5–8 nm), with high magnetization saturation, and very high heat generation capability, were prepared by the polyol approach. The MNPs were embedded in a dual pH- and temperature-responsive poly (N-vinylcaprolactam-co-acrylic acid) to provide stimuli-responsive MNCs with LCST ~40 °C at pH 5.5 and ~45 °C at pH 7.4. Remarkably, these MNCs turned out to be highly effective heat mediators with the SAR value of 357.0 W g^−1^_(Fe)_ at a frequency and amplitude suitable for clinical trials (*f* =183 kHz and *H_AC_* =17 kAm^−1^), with excellent drug loading capacity (EE% up to 98% for the sample B^+DOX^_7.4_) and promising stimuli-triggered drug release functionality. The latter was much more significant at pH = 4.5 and at 48 °C when an almost complete release of the conjugated drug (98.8%) was observed after 72 h of hyperthermia treatment (standard heating). Indeed, the dual pH- and temperature-responsive nature of the MNCs led to an intense release of the drug at a mimicked acidic tumor compartment upon application of an AMF, while a negligible amount of drug was released at physiological pH and body temperature, verifying that the drug was efficiently retained in the MNCs’ platforms and released in the response of exogenous/endogenous stimuli only. Moreover, drug release experiments when applying an on-off switchable AMF, showed that when the magnetic field is ‘on’, a controlled on-demand drug release occurred, while no release was observed when the AMF was switched ‘off’. Thus, these promising results demonstrated that our smart MNCs can be employed as an ‘on–off’ drug delivery system that is able to increase the therapeutic efficacy and reduce adverse side effects of chemotherapeutics. Finally, the developed MNCs revealed low cytotoxicity (10% after 6 h of incubation at 0.1 mg mL^−1^ of Fe_3_O_4_ MNPs) and DOX internalization into melanoma cells and, more efficiently, in breast cancer cells. In addition, a significant viability reduction is observed as the result of the combined effect of AMF field application and of the acidic pH of the endosomal compartment.

We can thus conclude that these MNCs are effective tools for tumor therapy by controlled properties, including highly efficient magnetic hyperthermia and magnetothermally/pH-controlled drug release at the right time and concentration by activation through external/internal stimuli. In the near future, a deep in vivo investigation will be performed to assess the efficacy of our MNCs as a new drug nanocarrier for tumor therapy, their biological fate, and the residence time in the body.

## Figures and Tables

**Table 1:** Magnetic properties of Fe_3_O_4_ MNPs before and after polymer coating. The experimental M_S_ has been approximated to the high field (50 kOe) value. Data are normalized to the amount of inorganic material.

Sample	M_S_ (emu g^−1^) 300 K	M_S_ (emu g^−1^) 10 K	M_R_ (emu g^−1^) 10 K	Coercivity (Oe) 10 K
Fe_3_O_4_	76	84	28	300
Fe_3_O_4_@Polymer	77	80	27	150

**Table 2:** Encapsulation efficiency (EE) and drug loading capacity (DLC) of samples A^DOX^ and B^DOX^ in buffer phosphate 10 mM at pH = 7.4 and 5.5 with different amount of MNPs (wt %) and DOX.

Samples	MNPs (wt %)	Polymer (wt %)	DOX (mg mL^−1^)	EE (%)	DLC (µg mg^−1^)
A^DOX^_7.4_	0.14	61	0.225	90	53.3
B^DOX^_7.4_	0.37	70	0.225	92	16.5
A^DOX^_5.5_	0.14	61	0.225	45	26.7
B^DOX^_5.5_	0.37	70	0.225	53	9.6
A^+DOX^_7.4_	0.14	61	0.375	96	91.1
B^+DOX^_7.4_	0.37	70	0.375	98	28.9

## Supplementary Materials

The following are available online at https://www.mdpi.com/article/10.3390/nano12030303/s1, Synthesis of PVCL-co-PAA; characterization of PVCL-co-PAA including FT-IR spectra, GPC characterization, ^1^H-NMR analysis of copolymer, and turbidimetric analysis; morphology of Fe_3_O_4_ MNPs; thermo- gravimetric analysis of sample B; temperature kinetics of Fe_3_O_4_ MNPs dispersed in DEG under application of an AMF at various *f* and *H*_0_; temperature kinetics of MNCs before and after drug loading at pH = 7.4 and pH = 5.5; LCST measurement of samples B_7.4_ and B_5.5_ by DLS analysis; temperature kinetics of B^DOX^_7.4_ and B^DOX^_5.5_ under an ‘‘intermittent’’ AMF (*f* ~183 kHz and *H*_0_ ~17 kAm^−1^); DLS data of MNCs and DOX-MNC suspension in DMEM supplemented with FBS [70,71].
