# Supplementary material for: Smart Magnetic Nanocarriers for Multi-Stimuli On-Demand Drug Delivery
Source: Nanomaterials (Basel). 2022 Jan 18;12(3):303. doi: 10.3390/nano12030303 (PMC8840331; doi:10.3390/nano12030303)
Supplement: Supplementary file 1 [file nanomaterials-12-00303-s001.zip › DONEupdate2-nanomaterials-1429042-supplementary/DONEupdate2-nanomaterials-1429042-supplementary.pdf]

nanomaterials

Supplementary Materials

Smart Magnetic Nanocarriers for Multi-Stimuli On-Demand Drug Delivery

Parisa Eslami [1], Martin Albino [1,2,*], Francesca Scavone [3], Federica Chiellini [4,†], Andrea Morelli [4], Giovanni Baldi [5], Laura Cappiello [5], Saer Doumett [5], Giada Lorenzi [5], Costanza Ravagli [5], Andrea Caneschi [1,2], Anna Laurenzana [3] and Claudio Sangregorio [2,6,*]

[1] INSTM and Dipartimento di Ingegneria Industriale—DIEF, Università degli Studi di Firenze, 50139 Sesto Fiorentino, Italy; parisa.eslami@unifi.it (P.E.); andrea.caneschi@unifi.it (A.C.)
[2] INSTM and Dipartimento di Chimica, U. Schiff, Università di Firenze, 50019 Sesto Fiorentino, Italy
[3] Dipartimento di Scienze Biomediche Sperimentali e Cliniche Mario Serio, Università degli Studi di Firenze, 50134 Florence, Italy; francesca.scavone@unifi.it (F.S.); anna.laurenzana@unifi.it (A.L.)
[4] INSTM and Dipartimento di Chimica e Chimica Industriale, Università di Pisa, 56124 Pisa, Italy; andrea.morelli@unipi.it
[5] Ce.Ri.Col, Colorobbia Consulting S.R.L, 50059 Sovigliana-Vinci (FI), Italy; baldig@colorobbia.it (G.B.); cappiellol@colorobbia.it (L.C.); doumetts@colorobbia.it (S.D.); lorenzig@colorobbia.it (G.L.); ravaglic@colorobbia.it (C.R.)
[6] ICCOM-CNR, 50019 Sesto Fiorentino, Italy
* Correspondence: martin.albino@unifi.it (M.A.); csangregorio@iccom.cnr.it (C.S.); Tel.: +39-055457-3270 (C.S.)
† To the memory of Prof. Federica Chiellini, a valued colleague and a dear friend of most of us.

S.1. Synthesis of PVCL-co-PAA

Polymerization was performed in dioxane as a solvent and a molar ratio of (NVCL/AA, 4:1) was selected, as it yields a polymer with a sharp LCST above 40 °C that can be easily reached through magnetic hyperthermia for controlled drug release. For this purpose, PVCL-co-PAA copolymer was prepared by free-radical copolymerization of NVCL with AA in the presence of AIBN as initiator. The procedure used 1.0 g (80.0 mol %) of NVCL, 0.123 mL (20.0 mol %) of AA, and 2.0 mol % of AIBN dissolved in 12.0 mL of dioxane. The mixture was purged with nitrogen for 30 min and let to react in an oil bath at 70 °C for 24 h under nitrogen flow. Afterward, the solution was cooled to room temperature, and the copolymer was precipitated in diethyl ether at low temperature. The precipitation was carried out three times to remove unreacted material, and the product dried under vacuum at room temperature for 48 h. Further purification was performed by dialysis technique using a dialysis bag with MWCO of 14 kDa against distilled water for 24 h. After lyophilization, the polymer was stored at 4 °C.

S.2. Characterization of PVCL-co-PAA

^{1}H-NMR (Bruker 400 MHz NMR) spectroscopy was conducted using $CDCl_3$ as a solvent to confirm the polymerization reaction advancement and assign the polymer structure as shown in Figure. S1. The ^{1}HNMR spectra of copolymer exhibited peaks at 4.38 ppm (1H$_a$, –NCH–of the α position), 3.20 ppm (2H$_b$,–NCH_2–), 2.48 ppm (1H$_{a'}$, –HCO_2CH– of the α position), 2.34 ppm (2H$_c$, –$COCH_2$– of the caprolactam ring), 1.78 ppm (2H$_{d'}$, –CH_2– of the backbone), 1.53 ppm (2H$_d$, –CH_2– of the backbone), 1.44 ppm (6H$_e$, –CH_2– of the caprolactam ring).

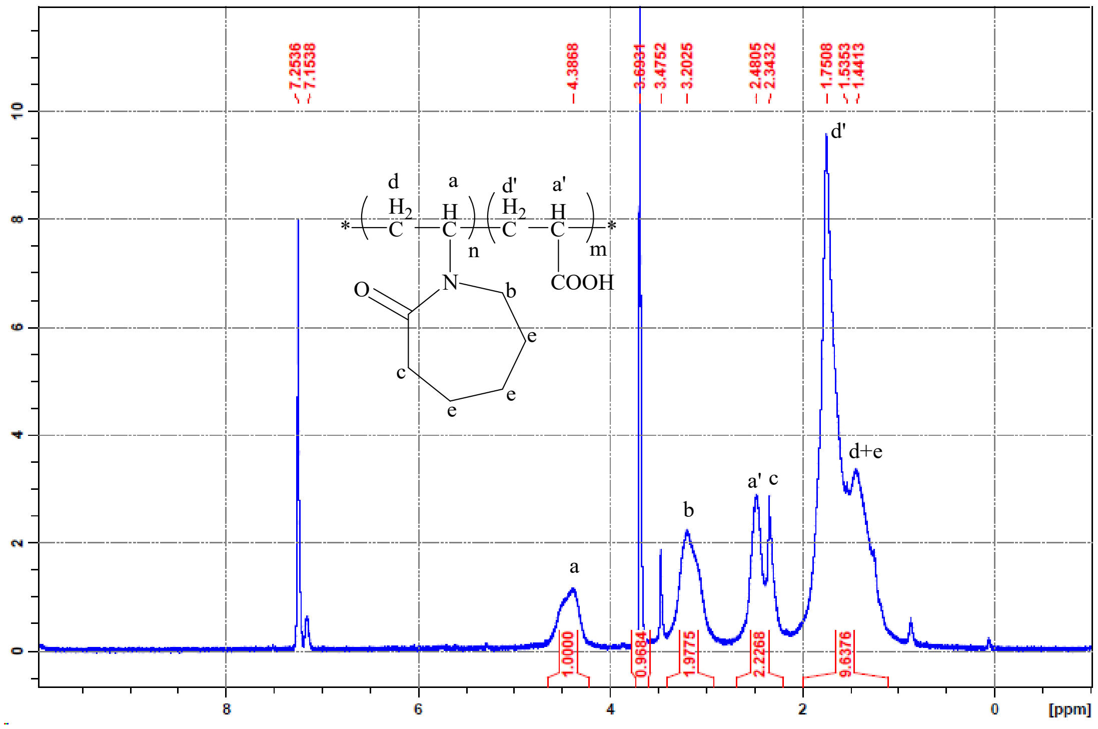

Figure S1. ^{1}HNMR of copolymer

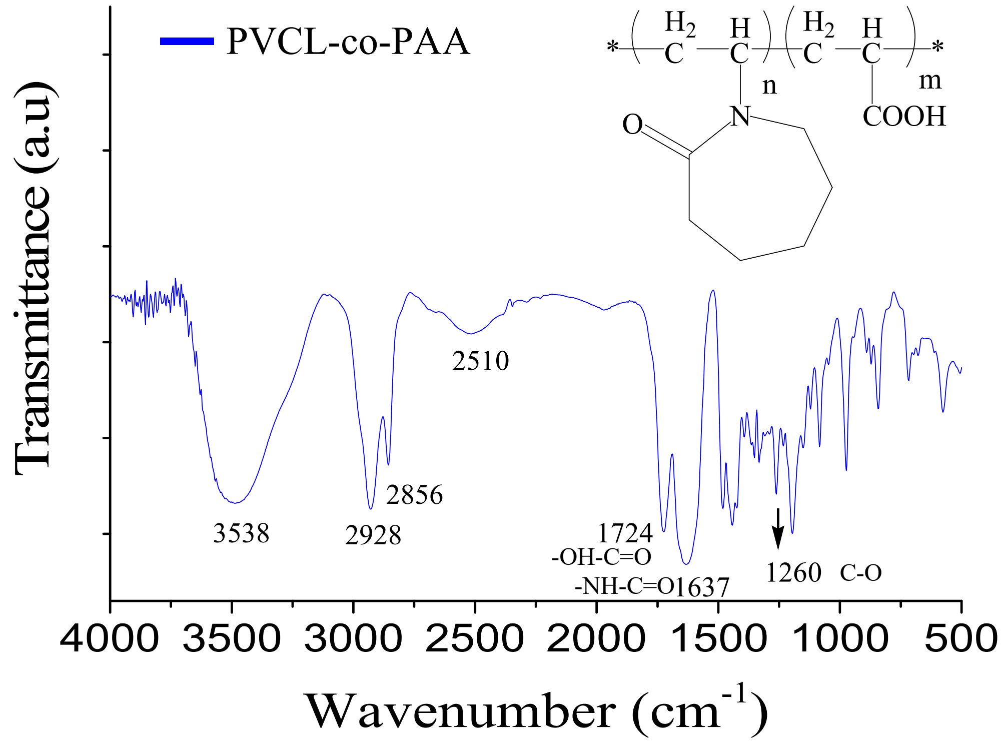

Figure S2. FTIR spectrum of PVCL-co-PAA

The success of the polymer synthesis protocol was confirmed by FT-IR analysis. The spectrum of the copolymer (Figure. S2) shows the typical absorption peak of the amide carbonyl group (-CON-) in the PVCL domain at 1637 cm^{-1}. IR also reveals a peak at 1724 cm^{-1} caused by the stretching vibration of -COOH (carboxylic) groups in the AA domain, while the peak at 1260 cm^{-1} can be assigned to the

characteristic absorptions of its C-O groups. Moreover, a wide peak at 3538 cm^{-1} is attributed to the absorptions of OH groups of absorbed water from the air. The peaks at 2856 cm^{-1} and 2928 cm^{-1} reveal the presence of aliphatic C-H groups in the structure of polymers. A specific peak at 2510 cm^{-1}, besides, is assigned to the internal hydrogen bond formation between carboxylic acid groups in the polymeric chain.

The molecular weight (M_n = 271890 g mol^{-1}, M_w = 402397.2 g mol^{-1}) and the molecular weight distribution (M_w/M_n = 1.48) of the copolymer were measured by Gel Permeation chromatography (GPC, Figure. S3). GPC analysis of PVCL-co-PAA was carried out with a Waters 600 model equipped with a Waters 410 Differential Refractometer and two Ultrahydrogel TM linear 6–13 μm columns (7.8 × 300 mm) at 25 °C. Mobile phase, constituted by water (HPLC grade), was eluted at a flow rate of 1.0 mL min^{-1}. Polyethylene oxide standards were used to obtain a calibration curve (M_n range 560–879000 g mol^{-1}).

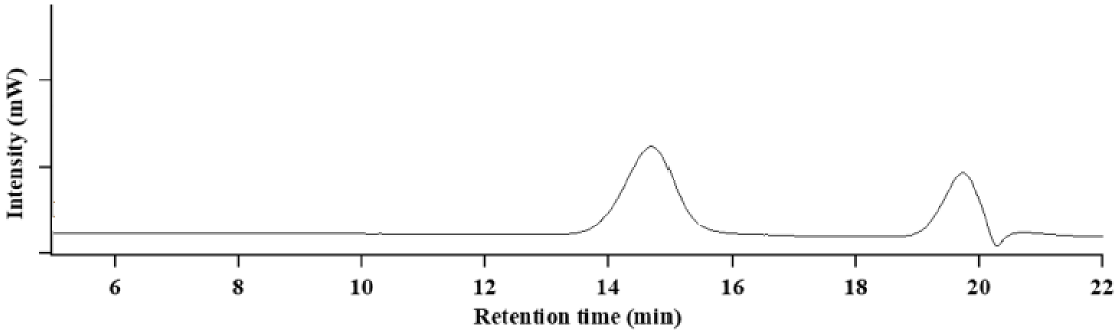

Figure S3. GPC chromatogram of PVCL-co-PAA

LCST of PVCL-co-PAA solution was determined by recording the transmittance of 1.0 mL copolymer solutions (6.0 mg mL^{-1}) prepared in buffer phosphate (10 mM, pH 7.4 and pH 5.5) with a Varian Carry 4000 UV 153 spectrophotometer coupled with a temperature controller. Before each measurement, the sample was left to equilibrate at the desired temperature for 5 min. The LCST values were obtained by monitoring change in transmittance as a function of temperature at 570 nm wavelength during the heating cycle (heating rate was 5 °C min^{-1}). The LCST value was taken as the inflection point of the transmittance curve versus temperature. Thus, corresponding to turbidity analysis of PVCL-co-PAA solution, LCST ~ 39 °C at pH = 5.5 and LCST ~ 43 °C at pH = 7.4 was obtained.

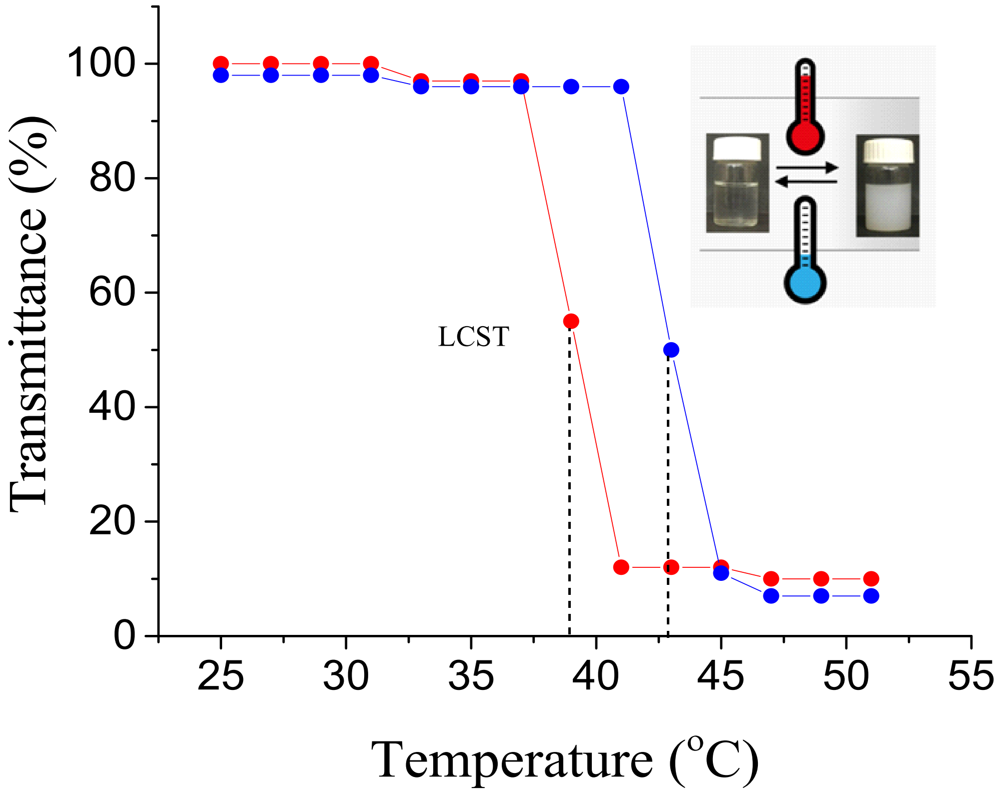

Figure. S4. Transmittance vs temperature curves for the PVCL-co-PAA copolymer in buffer phosphate 10 mM (6.0 mg mL^{-1}) at pH = 5.5 and pH = 7.4, determined by UV-Vis spectroscopy.

According to transmission versus temperature curves displayed in Figure. S4, in both cases the transmission sharply reduced when the temperature was raised above the LCST of the copolymer, since polymer undergoes a hydration/dehydration transition in the aqueous medium upon heating, resulting in a water solubility decrease. Thus, the polymer solution above the LCST is cloudy, whereas below the LCST appears as a clear and homogeneous solution.

Since acrylic acid (AA) chains in the polymeric backbone are strongly pH-sensitive, the LCST value of the thermo-responsive PVCL-co-PAA is pH-dependent. The double pH and temperature-responsive behavior of PVCL-co-PAA in aqueous/buffer solution can be explained considering that at T > LCST copolymer can form micellar structures [1], as schematized in Figure S5a. That means, at pH = 7.4 and T > 43 °C, the PVCL segment is insoluble in an/a aqueous/buffer solution, and it forms the core of a micelle with PAA forming the corona [2]. At pH = 5.5, when the temperature is raised above LCST, the formation of large aggregates occurs due to increasing insolubility of both protonated PAA corona and PVCL micellar core (Figure. S5b) resulting in a lower LCST value (~ 39 °C). These observations demonstrate PVCL-co-PAA copolymer, containing temperature- and pH-responsive segments self-assembled in water in response to both temperature and pH changes, is a suitable drug delivery system for remotely controlled drug release under hyperthermia performance and tumor acidic pH.

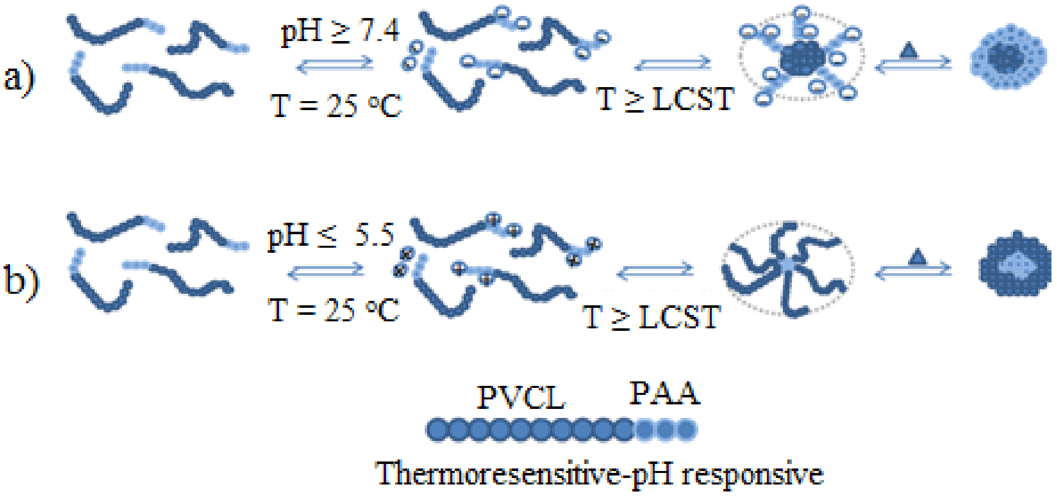

Figure S5. Dual pH and temperature-responsive behavior of PVCL-co-PAA solution

S.3. Morphology of Fe_3O_4 MNPs

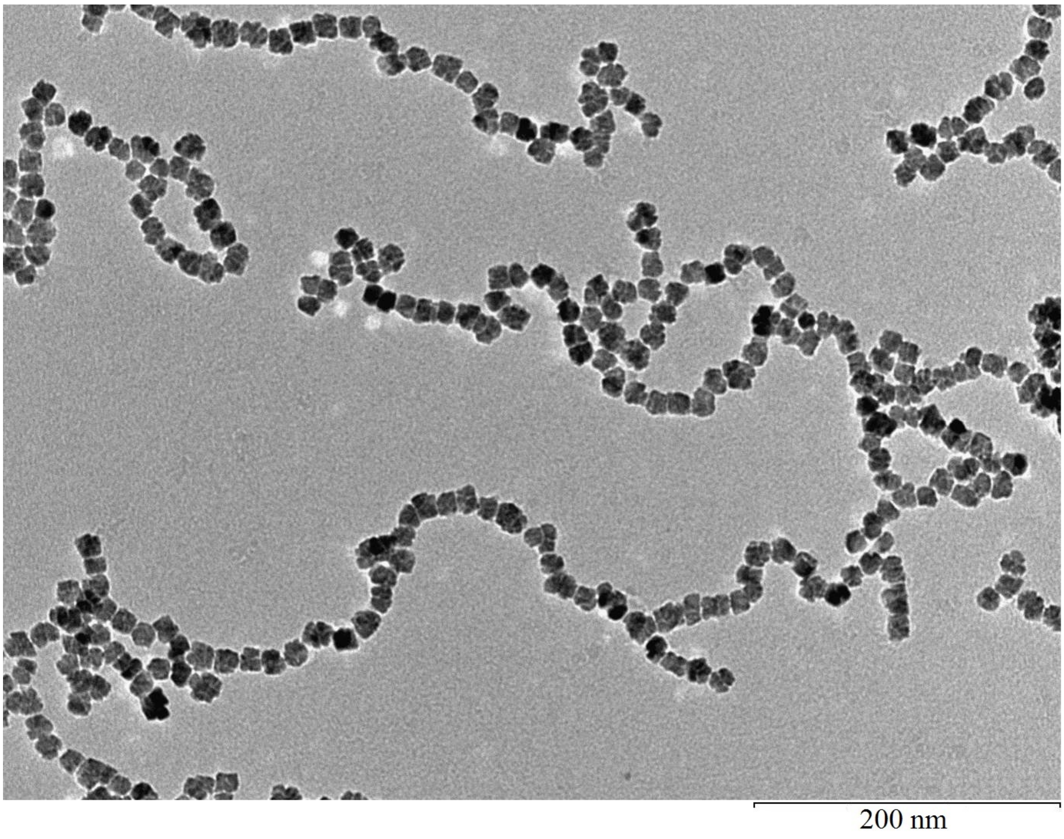

Figure. S6. TEM image of Fe_3O_4 MNPs

S.4. Thermal gravimetric analysis of sample B

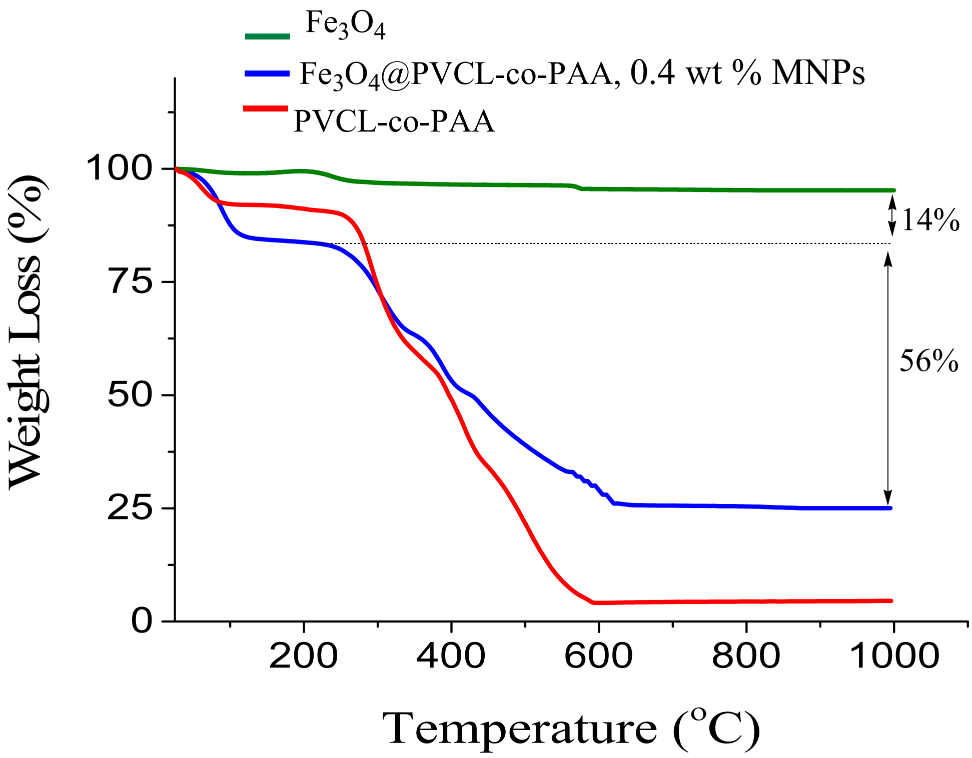

Figure S7. Thermogravimetric analysis of uncoated MNPs (green line), PVCL-co-PAA functionalized Fe_3O_4 (sample B, blue line) and PVCL-co-PAA (red line). TGA curve of MNCs revealed two significant steps in weight loss: the first at 98 °C (~ 14 %) can be attributed to the evaporation of residual water adsorbed on the surface, while the second sharp mass loss (~ 56 %) occurs between 240 °C to 634 °C that corresponds to the thermal decomposition of the polymer (246 °C to 591 °C, red line)

S.5. Hyperthermia efficiency of MNPs

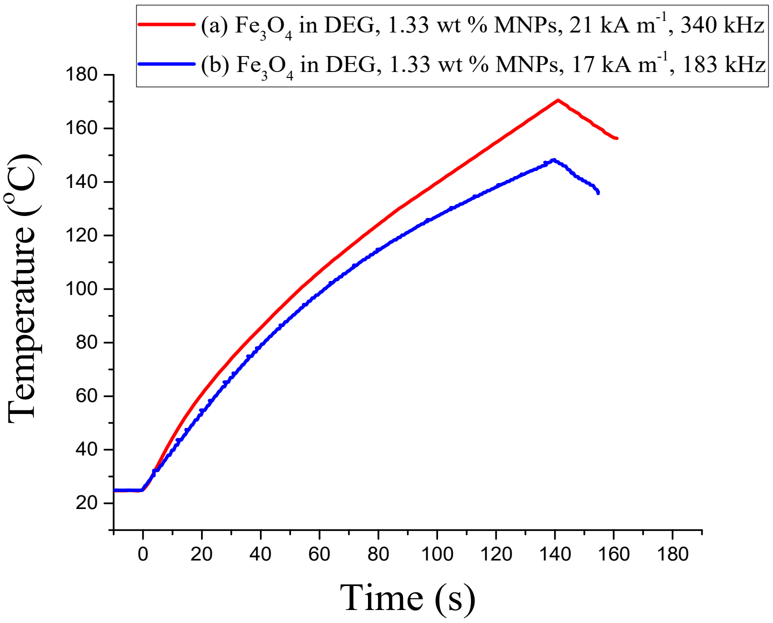

Figure S8. Temperature kinetics of Fe_3O_4 MNPs dispersed in DEG (1.33 wt % MNPs) acquired applying an alternating magnetic field of 21 kA m^{-1} amplitude (H_0) and 340 kHz frequency (*f*) (**a**) and 17 kA m^{-1} amplitude and 183 kHz frequency during140 s (**b**). Temperature variation versus time indicates the temperatures of ferrofluid (a) and (b) were raised to 170 °C and 147 °C respectively after 140 s of AMF exposure due to the excellent heat generation ability of flower-like Fe_3O_4 MNPs dispersed in DEG.

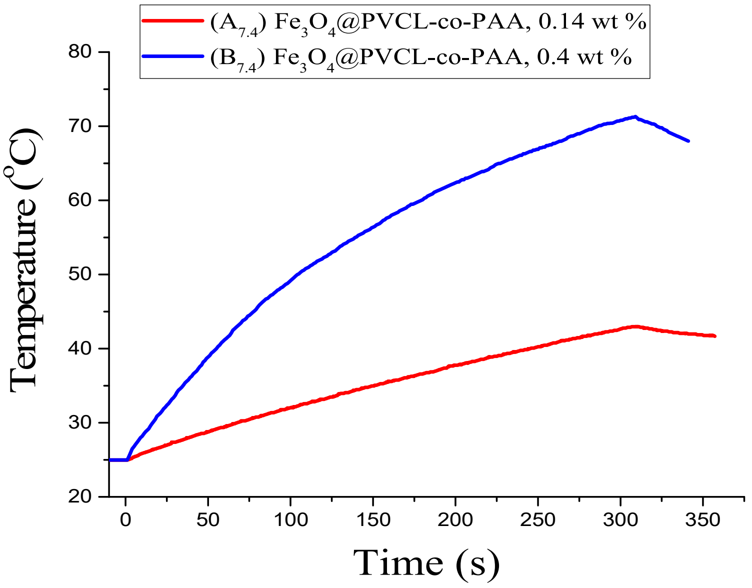

Figure S9. Temperature kinetics of MNCs dispersed in buffer phosphate 10 mM, pH = 7.4 (samples $A_{7.4}$ and $B_{7.4}$) acquired applying an AMF of H_0 = 17 kA m^{-1} and f = 183 kHz during 5 min. For both samples a large temperature increase is obvious, and the two ferrofluids reaching 43 °C ($A_{7.4}$) and 71 °C ($B_{7.4}$) respectively after 5 min of field application.

S.6. LCST measurement of samples $B_{7.4}$ and $B_{5.5}$ by DLS analysis

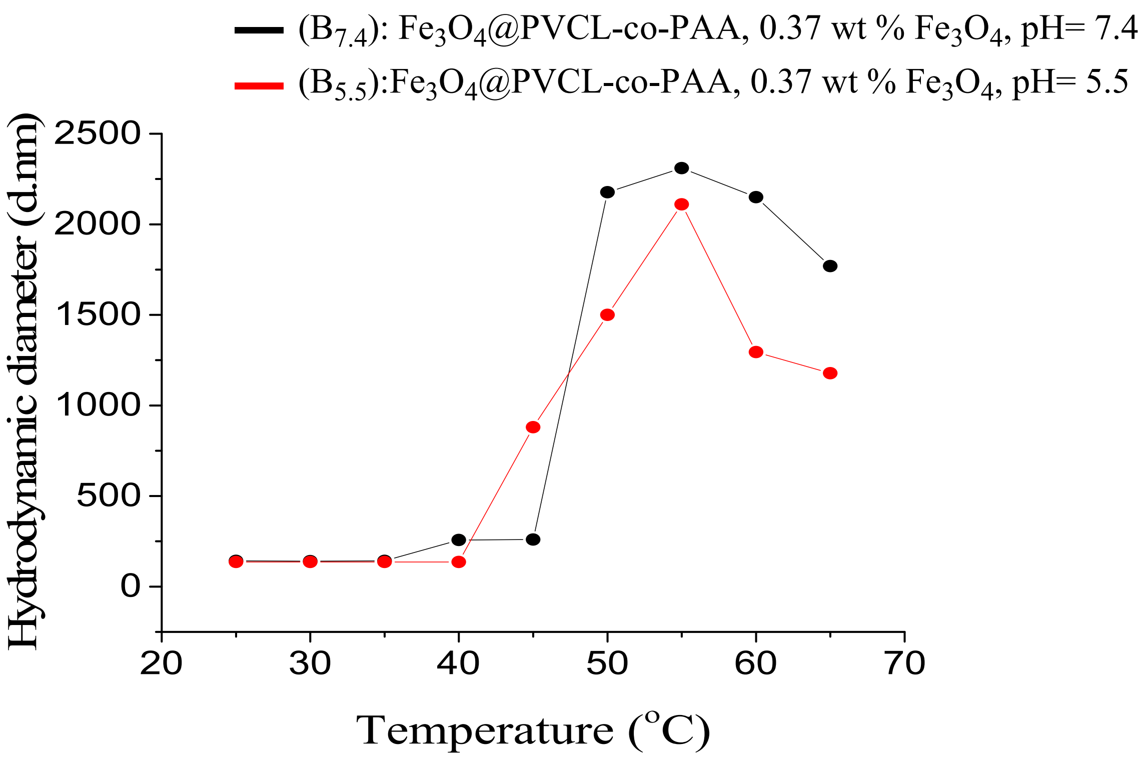

Figure S10. LCST of samples $B_{7.4}$ and $B_{5.5}$, determined by hydrodynamic size variation as a function of temperature according to DLS analysis. The hydrodynamic size of MNCs suspensions is highly temperature-dependent. The temperature at which the abrupt size increase occurred was taken as the LCST (~ 45 °C for $B_{7.4}$ and ~ 40 °C for sample $B_{5.5}$).

S.7. Hyperthermia efficiency of samples $A^{DOX}_{7.4}$ and $A^{DOX}_{5.5}$

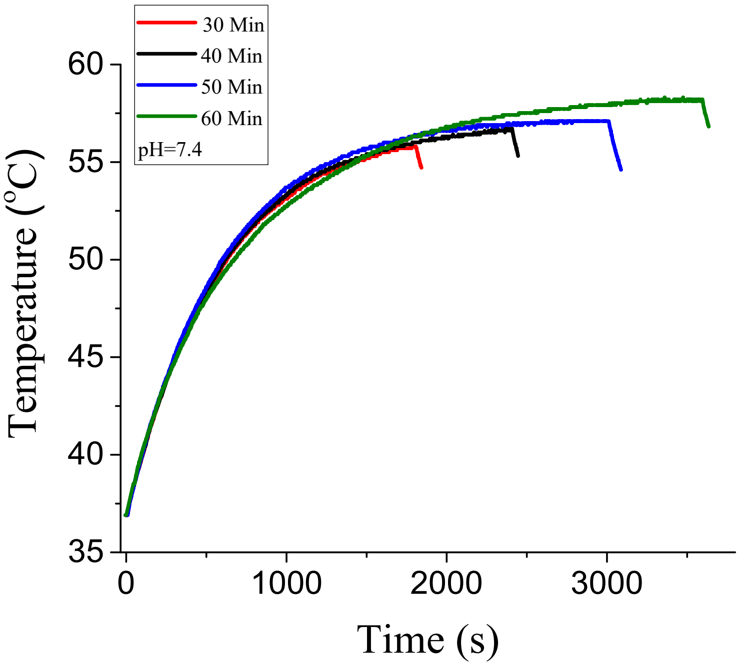

Figure S11. Magnetically induced thermal response curve of sample $A^{DOX}_{7.4}$ at different time intervals under exposure to AMF with f = 183 kHz and H_0 = 17 kAm^{-1}

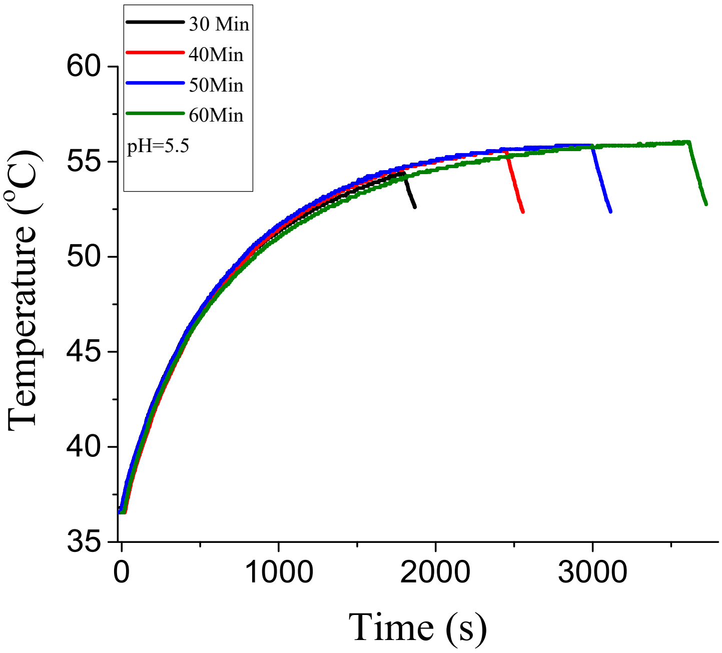

Figure S12. Magnetically induced thermal response curve of sample $A^{DOX}_{5.5}$ at different time intervals (AMF with f = 183 kHz and H_0 = 17 kAm^{-1})

S.8 A Smart hyperthermia with switchable drug release

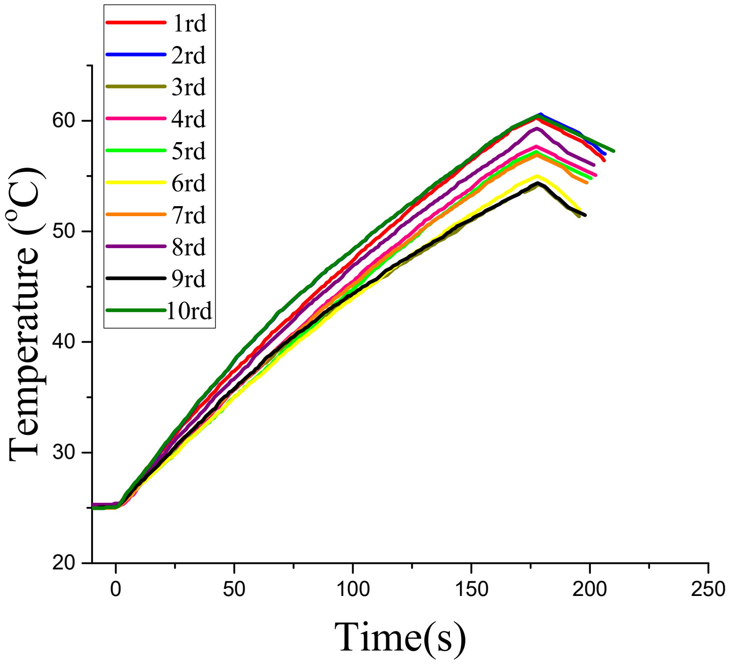

Figure S13. Magnetically induced thermal response curve of $B^{DOX}_{7.4}$ under "intermittent" AMF (f = 183 kHz and H_0 = 17 kAm^{-1}) for the overall time of 30 min (3 min, 10 cycles).

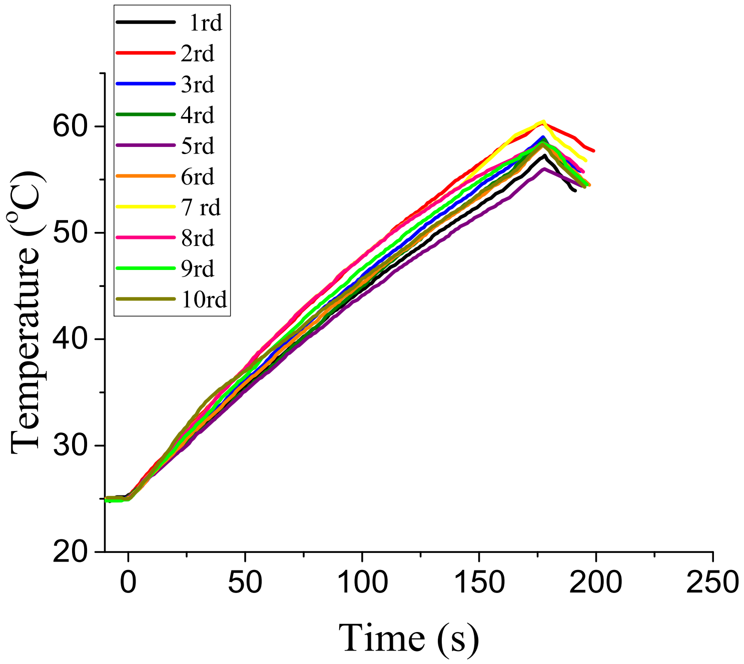

Figure S14. Magnetically induced thermal response curve of $B^{DOX}_{5.5}$ under "intermittent" condition of AMF (f = 183 kHz and H_0 = 17 kAm^{-1}) for the overall time of 30 min (3 min, 10 cycles)

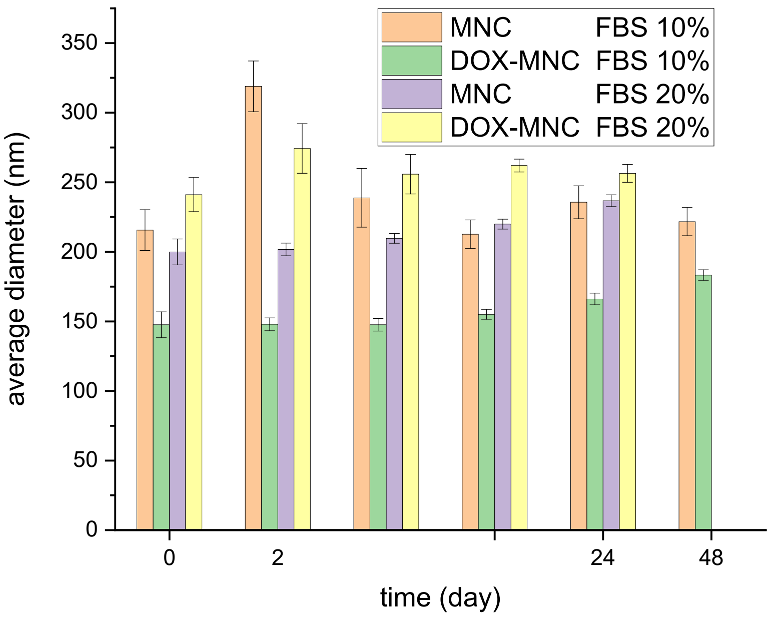

Figure S15. Time dependence of the avergae diameter estimated by DLS for MNC and DOX-MNC suspensions with 0.1 mg/mL^{-1} Fe_3O_4 and 1.0 μmol L^{-1} DOX concentrations in DMEM supplemented with FBS 10% (MNC, orange, and DOX-MNC, green) and 20% (MNC, purple, and DOX-MNC yellow).

References

1. Jiang, X.; Lu, G.; Feng, C.; Li, Y.; Huang, X. Poly(acrylic acid)-graft-poly(N-vinylcaprolactam): a novel pH and thermo dual-stimuli responsive system. *Polym. Chem.* **2013**, *4*, 3876-3884.
2. Schilli, C.M.; Zhang, M.; Rizzardo, E.; Thang, S.H.; Muller, A.H.E. A new double-responsive block copolymer synthesized via RAFT polymerization: Poly(N-isopropylacrylamide)-block-poly(acrylic acid). *Macromolecules* **2004**, *37*, 7861-7866.

© 2021 by the authors. Licensee MDPI, Basel, Switzerland. This article is an open access article distributed under the terms and conditions of the Creative Commons Attribution (CC BY) license (http://creativecommons.org/licenses/by/4.0/).
